# Astronomical spectra as language: Order-agnostic foundation model for low-SNR reconstruction and stellar parameter prediction

**DOI:** 10.1016/j.isci.2026.117165

**Published:** 2026-08-22

**Authors:** Jiacheng Xu, Xinrui Song, Yuyang Li, Cunshi Wang, Zhiwen Fu, Ali Luo, Jifeng Liu

**Affiliations:** 1 School of Low-altitude Science and Engineering, Shandong University, Weihai 264209, Shandong, China; 2 School of Astronomy and Space Sciences, University of Chinese Academy of Sciences, Beijing 100049, China; 3 National Astronomical Observatories, Chinese Academy of Sciences, 20A Datun Road, Chaoyang District, Beijing 100101, People's Republic of China; 4 Southern University of Science and Technology, 1088 Xueyuan Road, Shenzhen 518055, Guangdong, China

**Keywords:** large language model, spectroscopy, stellar parameter regression

## Abstract

Large-scale spectroscopic surveys contain numerous low-SNR stellar spectra that are difficult to analyze with traditional pipelines but remain valuable for Galactic archaeology, chemical-evolution studies, and population-level stellar characterization. We propose an order-agnostic foundation model that represents astronomical spectra as discrete tokens and learns intrinsic spectral correlations through arbitrary masking, enabling effective modeling of incomplete and noisy observations. The model is pretrained from scratch on physically motivated synthetic spectra in a self-supervised reconstruction task, and is then fine-tuned on observational LAMOST spectra to predict stellar parameters, with emphasis on metallicity ([Fe/H]). Experiments show that the model reconstructs low-SNR spectra with high fidelity and improves parameter estimation under noisy conditions. In the tested low-SNR setting, it achieves a metallicity prediction error of approximately 0.38 dex; even at extremely low-SNR values of about 1–2, it still provides useful information from spectra that are otherwise difficult to exploit.

## Highlights

• Digit-wise decimal quantization discretizes continuous spectral flux into tokens
• An order-agnostic transformer is proposed for astronomical spectral modeling
• Scratch pretraining learns spectral structure, then fine-tunes for stellar parameters

## Graphical abstract

**Figure:**
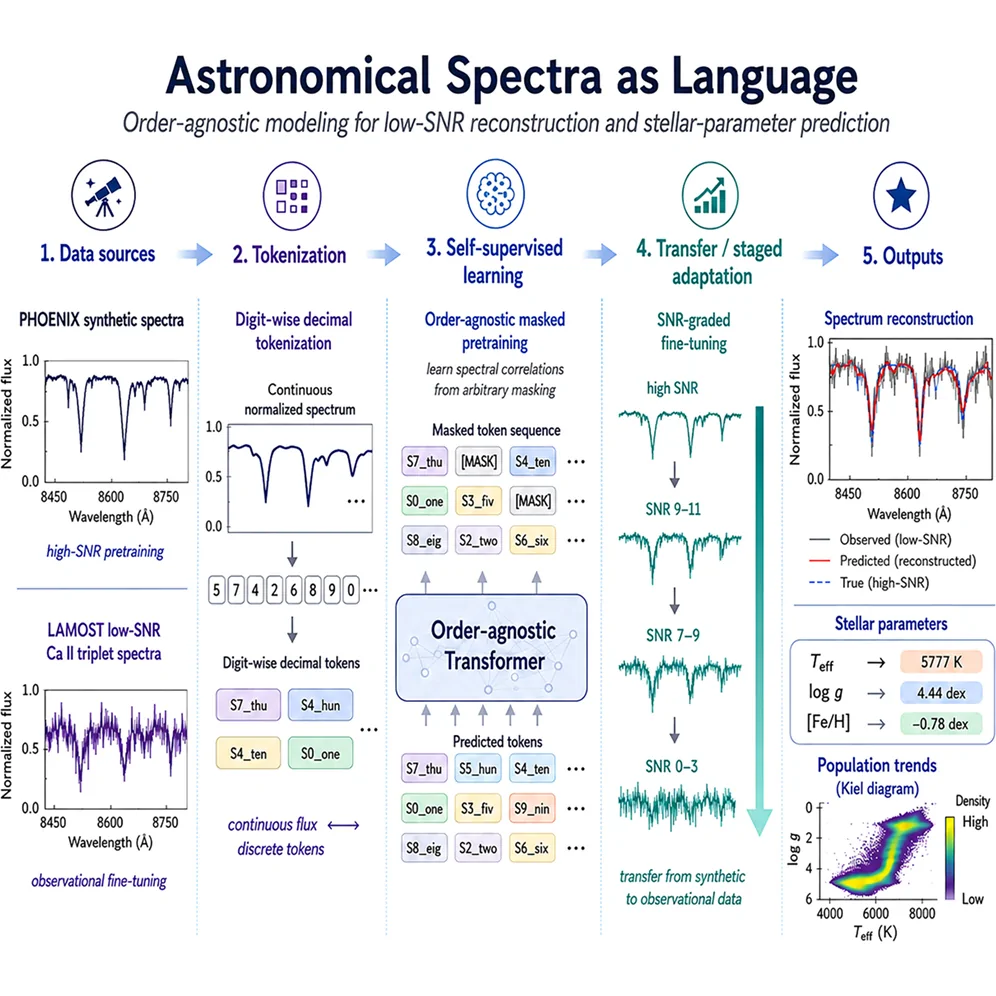

## Teaser

Astronomy; Network modeling

## Introduction

In the past two decades, astronomy has entered a new phase characterized by data-intensive surveys: from the digitized all-sky mapping pioneered by the Sloan Digital Sky Survey (SDSS; Almeida et al.^1^) to the reference design and planned data products of the Vera C. Rubin observatory’s legacy survey of space and time (LSST; Collaboration et al.^2^), survey scale, sampling cadence, and data multi-modality have grown in concert, transforming automated processing pipelines and machine-learning-based analysis from “useful auxiliaries” into indispensable infrastructure. Representative of large-aperture, multi-fiber spectroscopic facilities, the Large Sky Area Multi-Object Fiber Spectroscopic Telescope (LAMOST; Cui et al.,^3^ Zhao et al.,^4^ and Deng et al.^5^) has amassed on the order of ten million low-resolution stellar spectra, continually expanding the Milky Way stellar census and providing an unprecedented statistical foundation for studies of Galactic structure, chemical evolution, and time-domain astrophysics. This “large-facilities lead to big-data” landscape, in turn, poses an urgent methodological challenge: how to perform robust inference under low signal-to-noise ratio (SNR), heterogenous, and incomplete observations.

Traditionally, high-SNR spectra support precise stellar-parameter inference and detailed abundance determinations, whereas spectra with low-SNR are often difficult to exploit directly. As large low-resolution spectroscopic surveys have accumulated, the community has advanced two complementary approaches: data-driven label transfer, exemplified by *The Cannon*,^6^ a machine learning framework that uses a data-driven approach to assign stellar labels by learning from a large set of labeled spectra, and differentiable physics-based forward modeling or emulation that enables self-consistent fits, as in *The Payne*,^7^ a model that uses a physical stellar atmosphere model to predict stellar parameters through a forward-fitting approach. Together, these methods substantially reduce the cost of large-scale spectroscopic parameterization and show encouraging transferability in regimes of low-SNR.

In parallel with these representative frameworks, the methodological landscape for stellar-parameter estimation from low-resolution survey spectra has become increasingly diverse. Operational pipelines such as LASP and LSP3 demonstrate the continuing utility of classical or hybrid template-matching workflows at survey scale, while later data-driven systems extend this landscape through KPCA-based regression, low-SNR-oriented feature-engineering networks such as LASSO-MLPNet, recurrent deep models such as StarGRUNet, and CNN-based estimators tailored to very low-resolution metal-poor-star searches.^8^^,^^9^^,^^10^^,^^11^^,^^12^ Just as importantly, prior studies have shown that method assessment should not rely on a single aggregate score alone: standard regression errors, external-validation scatter, and diagnostics as functions of SNR and position in stellar-parameter space are all informative, because precision and bias vary non-uniformly across *T*_*eff*_, log *g*, and [Fe/H] regimes.^9^^,^^10^^,^^11^^,^^12^ This broader benchmark perspective is therefore essential for determining whether a new model offers merely competitive average accuracy or genuine robustness for low-SNR metal-poor applications.

Driven by rapid advances in computing, the transformer has established self-attention as a core operator for sequence modeling, overcoming the limitations of stepwise dependencies.^13^ The subsequent rise of masked modeling, such as Bidirectional Encoder Representation from Transformers and mask autoencoder (MAE), popularized a pretraining paradigm where reconstructing masked inputs yields robust structural representations even under noisy conditions.^14^^,^^15^ In this work, we propose a generative spectral modeling approach that predicts masked wavelength segments to learn intrinsic cross-band correlations in a self-supervised manner. We strategically optimize the training data selection to focus on the metal-poor domain, thereby tailoring the model to address the challenge of identifying extreme metal-poor candidates from low-SNR observations.

Viewed more broadly, sequence modeling in life sciences demonstrates that combining specialized tokenizers with self-supervised pretraining significantly enhances representation learning. Notable advances, such as the adoption of Byte-Pair Encoding in DNA modeling, highlight the effectiveness of discretizing complex data into linguistic units.^16^^,^^17^ This work supports the universality of our approach: we adapt this general paradigm to stellar spectra by designing a structure-preserving discrete tokenization to enable robust masked self-supervision.

Building on this serialization-driven perspective, our study addresses two critical limitations in current astronomical spectral analysis. First, existing machine-learning pipelines often depend on task-specific features or shallow architectures, failing to fully exploit the complex structure of modern heterogeneous surveys across the entire stellar parameter space. Second, a vast reservoir of low-resolution, low-SNR spectra remains largely unexploited, particularly for metal-poor star searches. To overcome these barriers, we present a universal paradigm for processing low-SNR data: a 130 million-parameter transformer framework trained from scratch. Our approach leverages masked diffusion and structure-preserving discrete serialization to learn intrinsic spectral correlations. Crucially, to enhance the model’s sensitivity to weak metallic features, we optimize the pretraining corpus by selectively sampling metal-poor templates. This data-centric optimization, combined with our redesigned vocabulary, results in a pretrained model that can be effectively finetuned for downstream tasks, supporting parameter inference and candidate screening even in high-noise regimes.

The complete data-processing procedures are described in data. Results for spectral inference and metrics are reported in the results section, while comparative experiments, strengths, limitations, and future work are discussed in the discussion section. The STAR Methods section (method details) details the design of our discrete tokenization grammar and our order-agnostic LLM architecture (model architecture), and introduces the pretraining protocol in pretraining, followed by the downstream physical-parameter regression in finetune.

## Results

**Figure 1:**
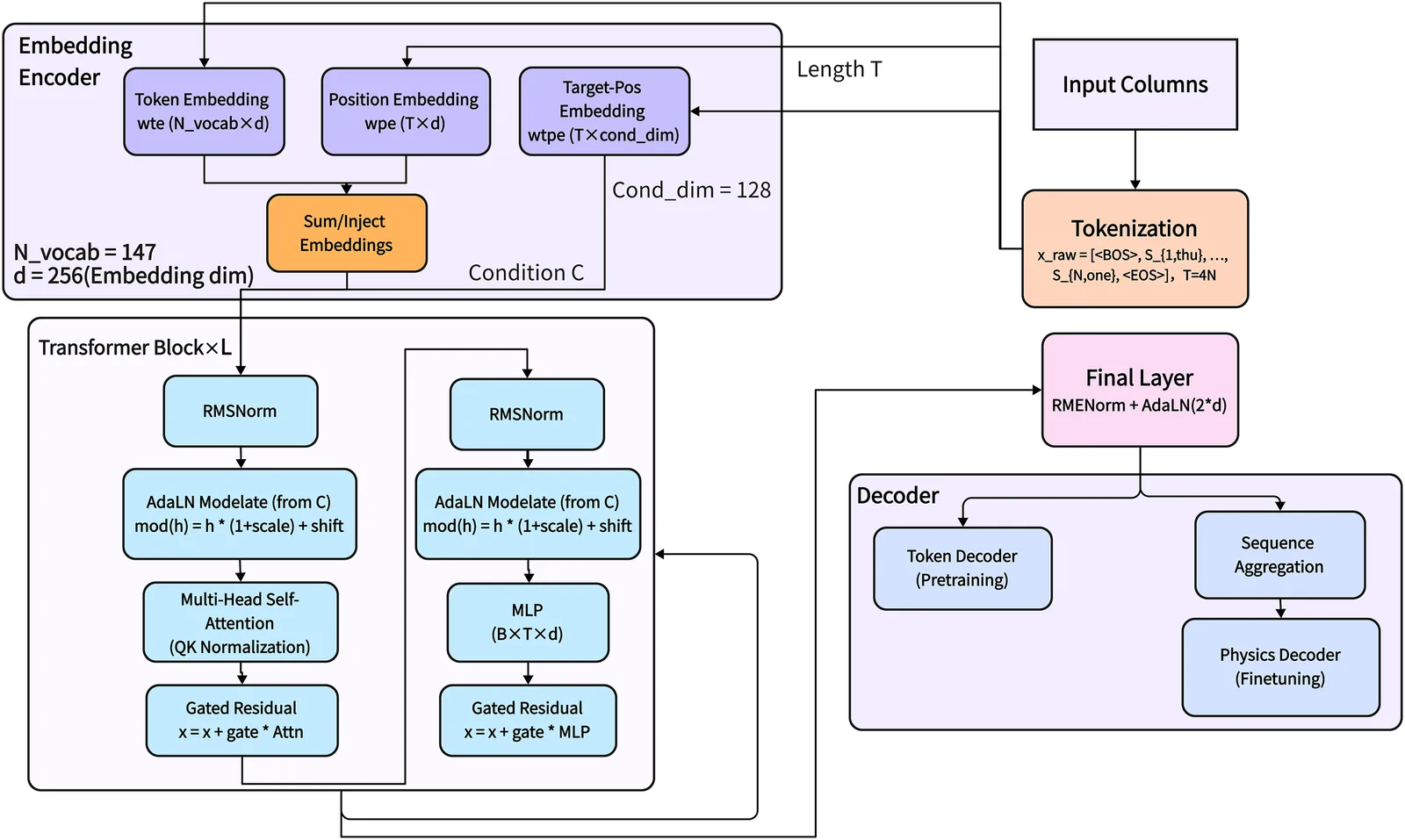
The model architecture diagram depicts the entire process from data input through discrete labeling, embedding, pretraining to finetuning

Under a unified validation split and metric protocol, we evaluate the model in both the position domain and the label domain. The position domain is assessed by the per-position on continuum-normalized spectra; the label domain reports Root Mean Square Error (RMSE) in the physical space for *T*_*eff*_, log *g*, and [Fe/H]. The aggregate score Total is the standardized (*Z* score) summary defined in the STAR Methods. The model architecture is shown in Figure 1.

### Phase I (3,030 samples, synthetic; pretraining)

With dense sampling and noise-free synthetic spectra, one full pretraining pass achieves a validation loss of 0.0936 and Flux-RMSE = 1556.25, which serves as the position-domain reconstruction baseline under ideal conditions.

Training dynamics are summarized in supplemental information; we use the checkpoint attaining the minimum validation loss for downstream finetuning.

During pretraining, the training loss decreases monotonically, while the validation loss steadily drops and experiences a distinct step-down around 33,000 steps, exhibiting only slight fluctuations thereafter.

**Figure 2:**
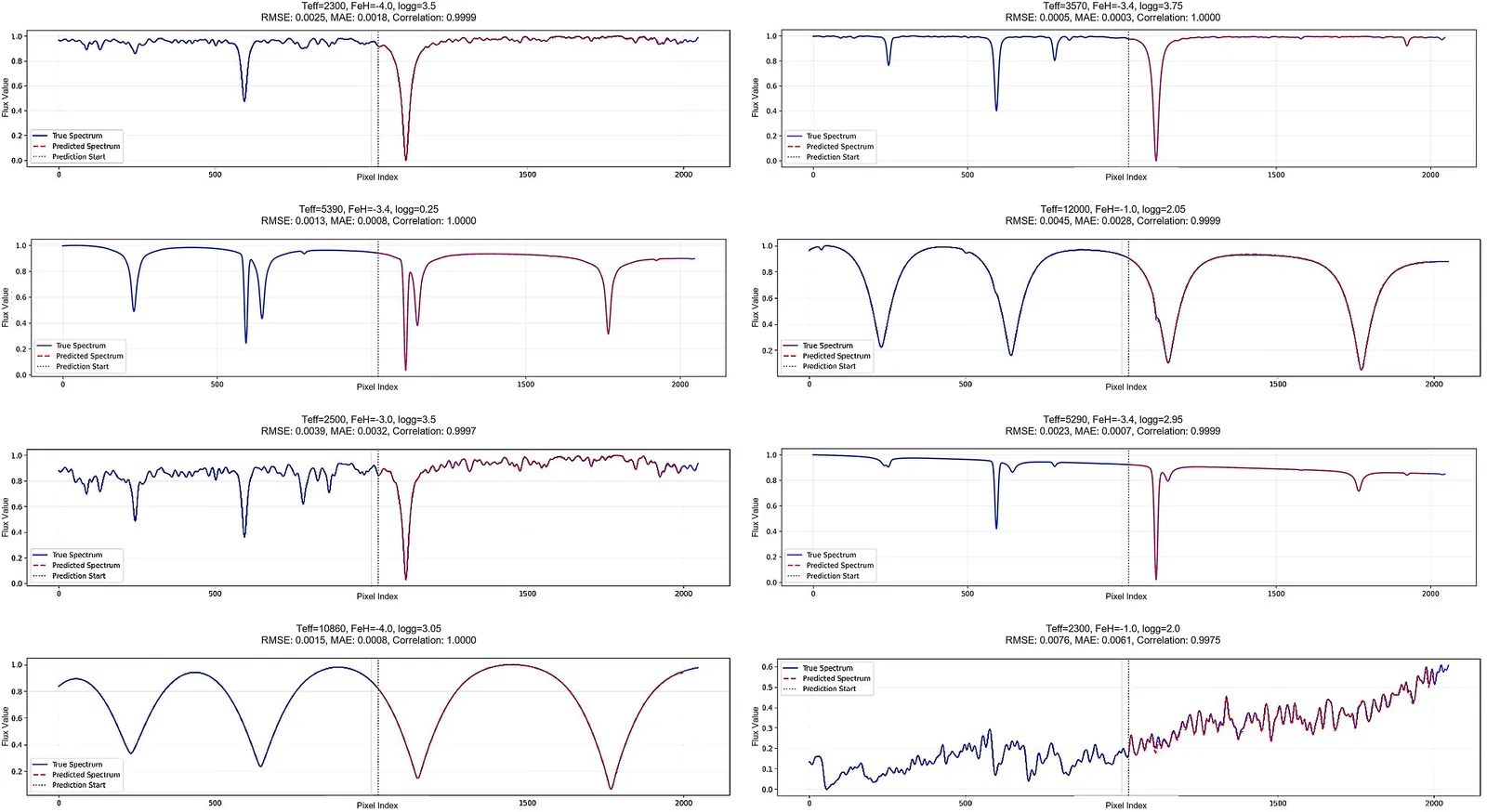
Pretraining inference on high-SNR synthetic spectra In each image, the left half of the spectrum is provided as condition and the model predicts the right half. Blue: given half; red: model prediction overlaying the ground truth. The dashed vertical line marks the prediction boundary. Image headers display per-example RMSE, MAE, and correlation; across the shown cases, the prediction error remains low and both line cores and continuum trends are preserved, indicating that pretraining has captured salient flux patterns and modes.

Figure 2 further provides a qualitative view of the half-spectrum forecasting task on high-SNR synthetic spectra. The model reconstructs missing wavelength segments with generally good agreement. The generated spectra show close agreement with ground truth across both continuum and line-dominated regions, maintaining structural continuity without introducing spurious oscillations. These results indicate that the pretrained backbone has learned the key flux patterns and mode structure of the spectra, including line cores, wings, and broad continuum trends, rather than merely fitting local noise.

### Phase II (3,030-position synthetic spectra with full finetuning)

After the spectral-pretraining stage on 3,030-position high-SNR synthetic spectra, we attach a lightweight regression head and perform full-parameter finetuning on the same synthetic dataset to regress the three physical parameters. Owing to the well-initialized backbone, the optimization process converges rapidly in the label space: on the validation subset at step 7,000, we obtain an RMSE of ∼33 K in *T*_*eff*_, ∼0.037 dex in log *g*, and ∼0.026 dex in [Fe/H], with a total RMSE of 19 (Table 1).

**Table 1:** Full finetuning performance on 3,030-position high-SNR synthetic spectra

Split	Loss	*T*_*eff*_	Log *g*	[Fe/H]	Total RMSE
Validation subset	0.00052	32.94	0.04	0.03	19.02
Training (monitor)	0.02819	83.51	0.06	0.04	48.21

*T*_*eff*_, log *g*, and [Fe/H] denote the RMSE of the predicted effective temperature, surface gravity, and metallicity, respectively.

As shown in the finetuning procedure of supplemental information (see Figure S3), both training and validation losses and RMSEs drop steeply in the early steps and then settle into a low but stable plateau, without signs of divergence or overfitting.

These results indicate that, in the high-SNR synthetic regime, the pretrained backbone can support accurate three-parameter regression.

### Phase III (303-position observational spectra; SNR-graded full finetuning)

Starting from the pretrained and finetuned 3,030-position model, we perform *full-parameter* finetuning directly on 303-position observational spectra, adopting a curriculum in which the SNR range is gradually decreased. Concretely, we processed the data sequentially on two lower SNR subsets, with SNR ranging from [9, 11] to [7, 9], respectively. At each stage, we use the same input space, tokenizer, and sequence layout; only the observational data distribution and SNR range change. The corresponding validation metrics are summarized in Table 2. The corresponding results of curriculum fine-tuning at different SNR levels are summarized in the table.

**Table 2:** Summary of 303-position full finetuning under different SNR ranges

SNR range	Loss	*T*_*eff*_	Log*g*	[Fe/H]	Total RMSE
7–9	0.1021	621.48	0.41	0.38	358.81
9–11	0.0972	576.70	0.46	0.36	332.96

Column *T*_*eff*_, log *g*, and [Fe/H] report the RMSE of effective temperature, surface gravity, and metallicity, respectively.

The loss and total RMSE remain low and increase only moderately as SNR decreases, indicating that the model converges rapidly at each stage and maintains stable performance despite the progressively stronger noise.

Viewed together, the SNR-graded curriculum demonstrates that the masked-diffusion backbone, once adapted through decreasing- SNR 303-position finetuning, can be further refined to handle increasingly noisy observational spectra without catastrophic degradation. The smooth trend from the [9, 11] to the [7, 9] bins (Total ≈ 333 and ≈359, respectively) shows that label-domain errors grow sub-linearly with the drop in SNR, and remain within an astrophysically useful regime even for SNR ∈ [7, 9].

This SNR-graded full finetuning thus provides an effective route to transferring the pretrained model from high-SNR synthetic templates to low-SNR observational spectra, enabling regression of *T*_*eff*_, log *g*, and [Fe/H] directly from noisy 303-position spectra. Additionally, we have predicted the LAMOST LRS spectra with SNR ranging from 0 to 3 (see Table 3).

**Table 3:** Predictions for the LAMOST LRS Spectra

Obsid	*T*_*eff*, *pred*_ (K)	Log *g*_*pred*_ (dex)	[Fe/H]_*pred*_ (dex)
581105084	5,855	4.14	−0.50
298505004	5,797	4.22	−0.66
911103017	5,920	4.10	−0.99
200604202	6,004	4.06	−1.54
121704188	6,038	4.14	−0.78
991012075	6,006	4.08	−1.33
837808079	6,272	4.10	−1.06
685402002	6,197	4.03	−1.68
713811059	5,902	4.10	−1.19

### Phase IV (303-position observational spectra; ultra low-SNR inference and population-level sanity checks)

#### Population-consistency checks from ultra-low-SNR CaT inference

We apply the model to extremely low-SNR spectra restricted to the narrow CaII triplet (CaT) window, and perform joint inference of *T*_*eff*_, log *g*, and [Fe/H] for a large stellar sample. The distribution of SNR-z spectra for inferring unlabeled data can be seen in supplemental information. We then conduct physical consistency checks from both the overall distributions and the two-dimensional structures in the Kiel diagram.

#### Overall distributions

For the effective temperature *T*_*eff*_, the sample is dominated by F- or G-type main-sequence stars or subdwarfs, with a small extension toward cooler K-type and hotter A-type objects.

For the surface gravity log *g*, the bulk population remains dwarf-like (log *g* ≈ 4.1), while the low-log *g* and high-log *g* wings become visibly broadened; the Kiel diagram thus becomes closer to realistic stellar populations, and includes a small number of evolved stars and compact dwarfs. Under extremely low SNR, we also cannot exclude the possibility that some peculiar objects are spuriously projected into the high-log *g* region.

For metallicity [Fe/H], about 51% of the sample is inferred to be metal-poor with [Fe/H]<–1, with the main peak at [Fe/H]∈[ –1.4, –0.7], while retaining a small fraction of near-solar-metallicity contaminants. This metallicity portrait is consistent with the typical expectation that the CaT can serve as a metal-poor candidate selection feature.

#### Phenomenon analysis

**Figure 3:**
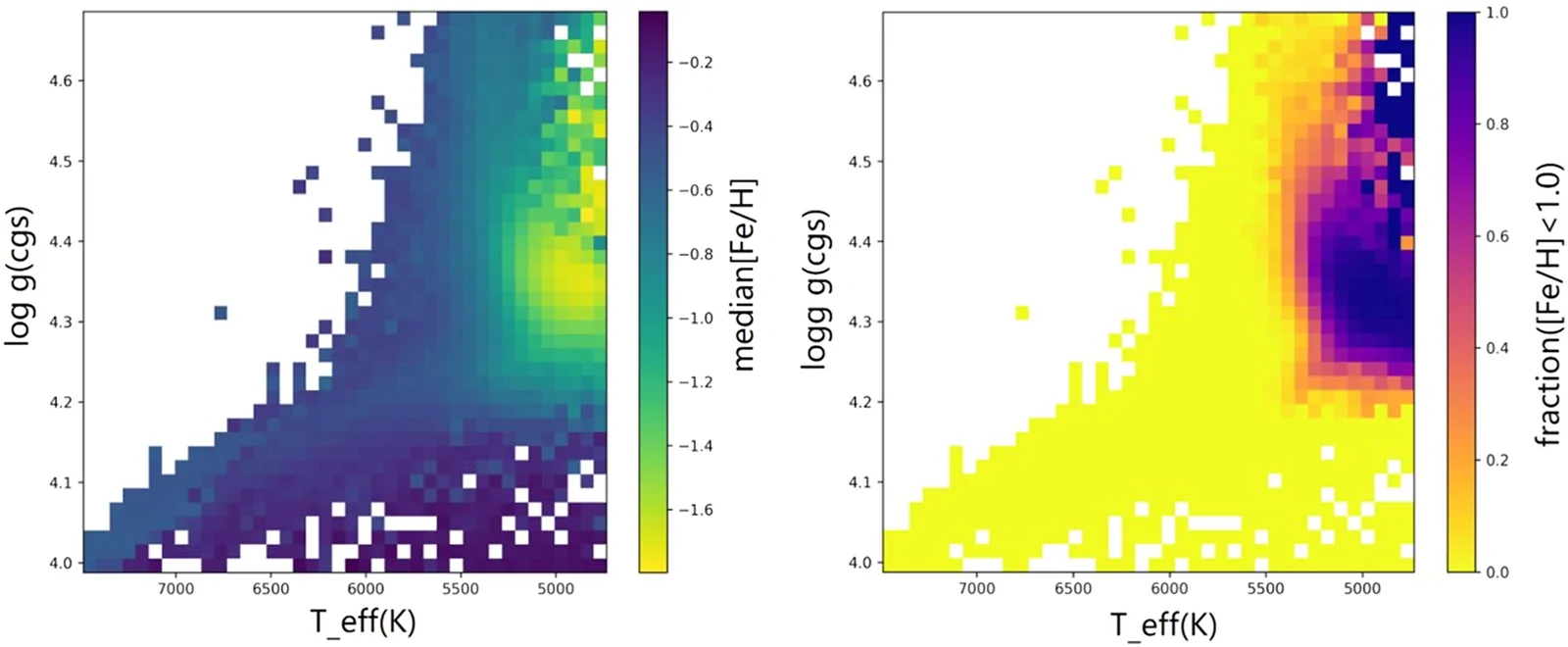
Two-dimensional population structure in the Kiel plane from ultra-low-SNR CaT area Left: The median metallicity [Fe∕H] across the (*T*_eff_ , log *g*) plane. Right: The spatial distribution of the metal-poor fraction, defined as Pr([Fe∕H] < –1), on the same grid.

On the (*T*_*eff*_, log *g*) grid, the median-[Fe/H] map exhibits a smooth gradient: the bottom-left region (higher *T*_*eff*_, lower log *g*) is more metal-poor, while the top-right region (lower *T*_*eff*_, higher log *g*) is relatively more metal-rich (see Figure 3). The Kiel diagram also shows a clear main-sequence band, where log *g* decreases slightly as *T*_*eff*_ increases.

**Figure 4:**
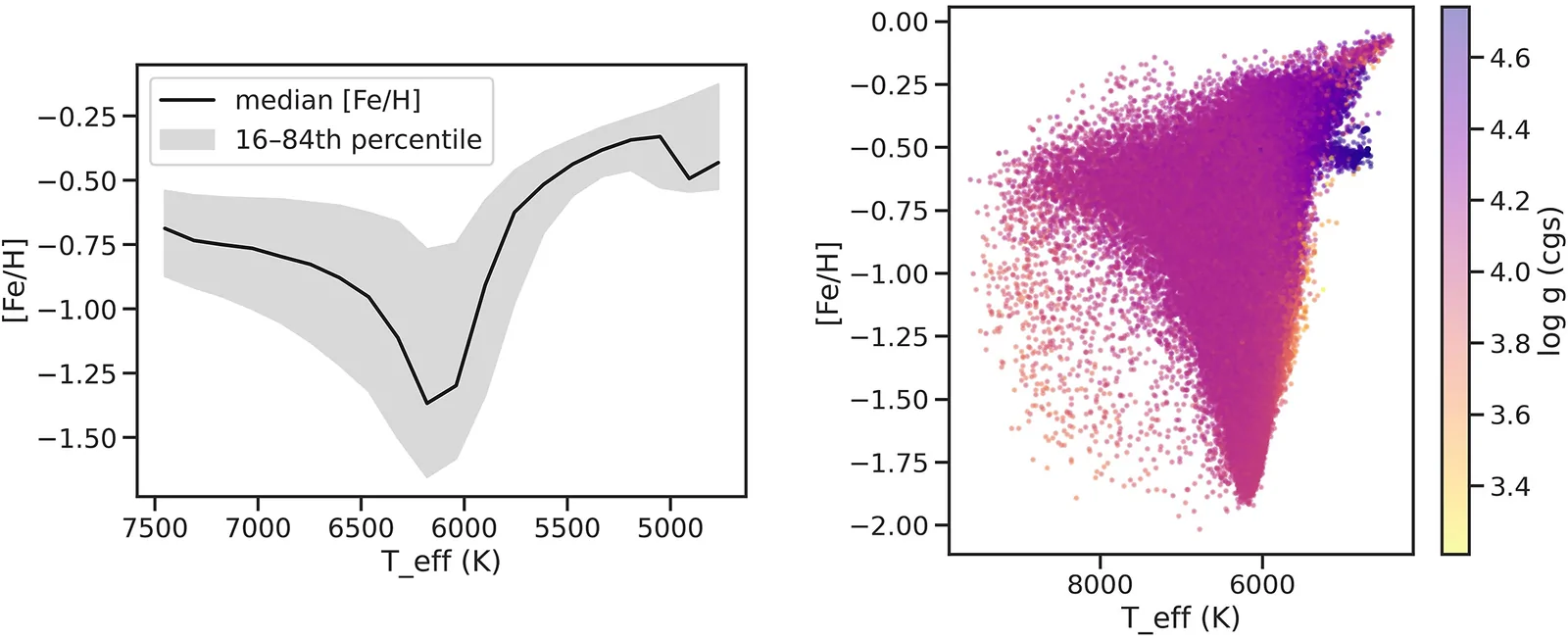
Population-level consistency checks from ultra-low-SNR CaT inference Left: The median metallicity [Fe∕H] as a function of *T*_eff_ , with the 16th–84th percentile interval. Right: The (*T*_eff_, [Fe∕H]) distribution with points colored by log *g*, illustrating the two-dimensional structure in the Kiel plane.

After binning by *T*_*eff*_, the median [Fe/H] decreases monotonically across *T*_*eff*_∼5,500 K to *T*_*eff*_∼6,400 K: at the cool end, the median metallicity is [Fe/H] ∼ –0.7, while near the hotter, turnoff-like regime the median drops to [Fe/H]∼ –1.4 (see Figure 4).

## Discussion

### Efficient prediction of low-SNR stellar parameters

Our experimental progression systematically validates the efficacy of the proposed framework across three distinct stages (see results).

In phase I, the pretraining stage enabled the model to learn intrinsic spectral features and morphologies, providing it with useful capabilities for spectral prediction and completion (see Figure 2). This result confirms that our structure-preserving tokenization and masked-diffusion architecture are highly suitable for modeling the complex nature of spectral data, laying a solid foundation for downstream regression tasks dependent on spectral structures.

In phase II, full-parameter finetuning on high-SNR spectra yielded precise parameter estimation. This successfully demonstrates that the downstream task can effectively leverage the structural priors and knowledge acquired during the pretraining phase to accurately map spectral features to physical labels.

In phase III, by adopting a curriculum learning strategy with progressively decreasing SNR, the model acquired a certain ability to infer stellar parameters from extremely low-SNR spectra. It still retains meaningful predictive performance in noise-dominated regimes (see Table 2), while preserving considerable physical utility and reference value.

### Error analysis on the Kiel diagram

#### Stability in the dwarf sequence

**Figure 5:**
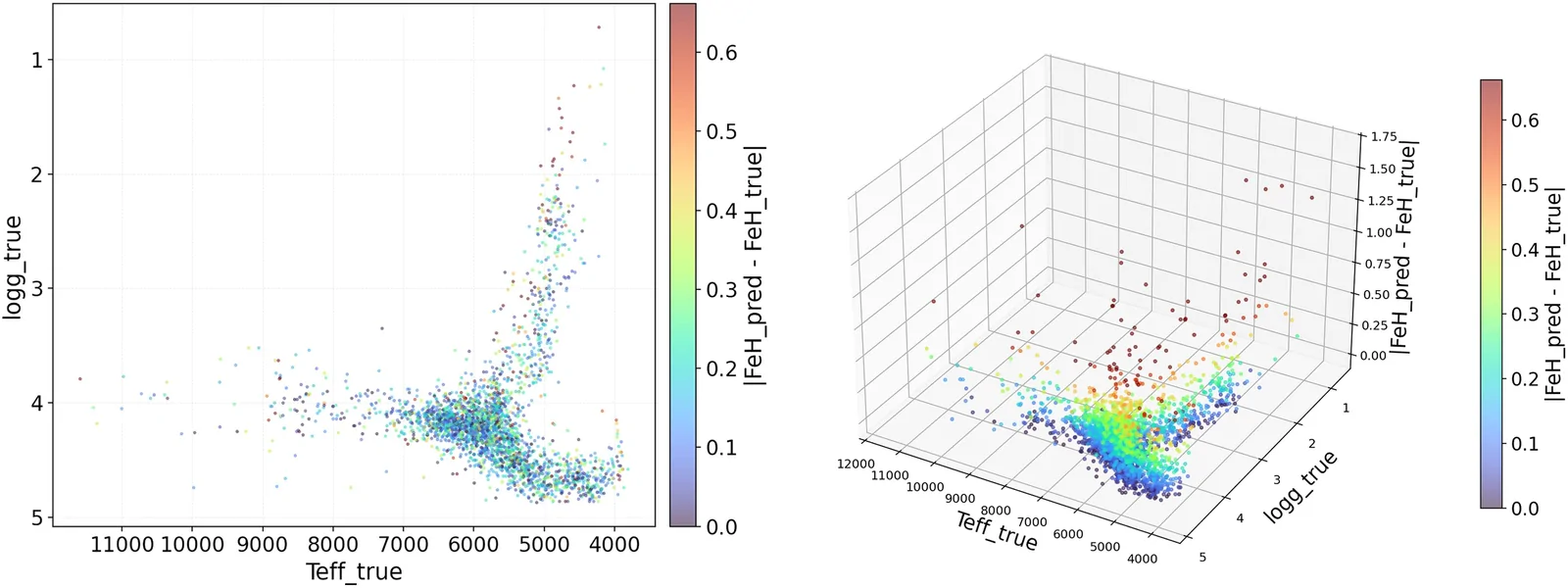
Metallicity prediction residuals projected onto the Kiel diagram The left image plots the test sample (*N* = 2,761) distribution in the *T*_eff_–log *g* plane, while the right image provides a three-dimensional perspective. The color gradient denotes the absolute prediction error |[Fe∕H]_pred_ – [Fe∕H]_true_ |, mapping model accuracy across the evolutionary parameter space.

Figure 5 maps the absolute metallicity prediction error (Δ[Fe/H]) across the physical *T*_*eff*_-log *g* plane.

The dwarf sequence (log *g* ∈ [4.0, 5.0]) exhibits robust stability. In the intermediate temperature regime (4,000 K ≤ *T*_*eff*_≤7,000 K), the residuals remain consistently low. Our model effectively extracts information from the target spectral area, leveraging line wings and continuum features prominent in high-gravity atmospheres to constrain main-sequence metallicity.

#### Consistency in evolved stars

In the low-gravity regime (log *g* < 3.5 dex), our model maintains reliable performance despite the distinct spectral morphology of giant stars. The residuals demonstrate a smooth continuity from the main sequence to the giant branch, confirming the framework’s capability to generalize across surface gravity variations without substantial loss of accuracy.

#### Performance in the high-temperature regime

A notable pattern is observed in the high-temperature domain (*T*_e*ff*_ ≥ 7,000 K), a region populated by hot dwarfs and blue giants where metallic absorption lines are intrinsically weak. Traditional methods often face difficulties in this regime due to the lack of strong spectral features. However, our proposed preliminary solution yields reasonably constrained predictions in this regime. By utilizing the intrinsic spectral correlations and global continuum trends learned during pretraining, the model yields reasonably constrained predictions, offering a viable approach for metallicity inference in these feature-scarce hot stars.

### Physical consistency and methodological efficacy at ultra-low SNR

The inference results on ultra-low SNR spectra provide a distribution-level sanity check for our proposed framework. By producing population-level structures that are broadly consistent with physically meaningful trends in noise-dominated data (SNR∈[0, 3]), we provide evidence supporting the three core design choices: structure-preserving tokenization, the masked-diffusion backbone, and the staged finetuning curriculum. Leveraging these architectural advantages, our model not only captures intrinsic spectral morphologies, as suggested by the reconstruction performance but also retains partial robustness in noise-dominated regimes. Consequently, it delivers physically plausible and scientifically valuable inference results even under conditions of extremely low SNR. It should also be noted that ensemble predictions preserve a reasonable overall structure rather than accurately restoring the values of *T*_*eff*_, log *g*, or [Fe/H] for every individual star; consequently, the prediction results will still be influenced by the learned prior and the compression of the metallicity distribution.

The inferred distribution in the Kiel diagram is broadly consistent with known stellar-population trends in the Milky Way: old, metal-poor thick-disk turnoff stars and younger, more metal-rich thin-disk dwarfs occupy different directions. Specifically, the inferred [Fe/H] distribution indicates that the vicinity of the main-sequence turnoff (higher *T*_*eff*_ with slightly lower log *g*) is more metal-poor, whereas cooler dwarfs with higher log *g* are somewhat more metal-rich.

The inferred Kiel-plane pattern is broadly consistent with the canonical Galactic picture at the population level. In particular, the region near the main-sequence turnoff, characterized by higher *T*_*eff*_ and slightly lower log *g*, appears more metal-poor, whereas cooler dwarfs with higher log *g* appear relatively more metal-rich. We therefore interpret this pattern as population-level consistency with known Galactic population trends, rather than as a complete identification of the underlying Galactic components or as evidence for accurate object-level parameter estimation in the SNR ≈ 1–3 regime.

This pattern confirms that the pretraining on high-SNR synthetic data, followed by the SNR-graded finetuning, has allowed the backbone model to learn the intrinsic manifold of stellar spectra. Instead of overfitting to noise, the model utilizes the learned priors to disentangle faint spectral features from the background, reproducing the well-known trend that hotter turnoff stars are more likely to belong to older, metal-poor populations.

Our analysis of the metallicity gradient shows that the uncertainty band (16th–84th percentiles) is not severely compressed. This indicates that the model is not simply pulling all hot stars toward a fixed [Fe/H], but is genuinely separating a more metal-poor population at the hot end. This behavior is consistent with the intended role of our digit-tokenization strategy. Unlike continuous regression which may collapse to the mean under high uncertainty, the discrete token representation preserves the probabilistic distribution of the flux. Even at moderate-to-low resolution and under low-SNR conditions where information content is vulnerable to noise and residual sky features, the model maintains a stable representation of the signal, providing suggestive evidence that it can still extract population information from noise-dominated CaT spectra. This suggests that even under the condition of SNR∈[0, 3], the model can still produce a physically plausible two-dimensional metallicity gradient. This provides a useful indication of the model’s potential and supports the feasibility of combining discrete representation with the generative pretraining objective.

Beyond statistical metrics, we further validate the physical plausibility of the model’s inference on the ultra low-SNR CaT spectra. First, the model identifies a stellar sample dominated by F- and G-type dwarfs and subdwarfs, with metallicities primarily in the range of −2.0≤[Fe/H]≤0.0, and approximately half of the sample having [Fe/H]< –1. This composition is broadly consistent with the average temperature and metallicity properties of metal-poor candidates selected via traditional CaT analysis. Therefore, judging from the distribution-level consistency with CaT-selected metal-poor candidates, the inference results provide a physically plausible sanity check under ultra-low-SNR conditions.

Second, regarding the one-dimensional distributions of *T*_*eff*_ and log *g* (visualized via histograms and Kernel density estimation), the results exhibit concentrated main peaks with smooth tails, notably devoid of unphysical spikes in extremely cool/hot or low/high gravity regimes. The number of extreme outliers (1,782 objects) is negligible compared with the total sample size and is confined to expectedly difficult regions, such as extremely hot or cool stars. This indicates that within the bulk-sample region, the model’s learned priors integrate well with the information extracted from the Ca II spectral features, avoiding widespread non-physical solutions.

**Figure 6:**
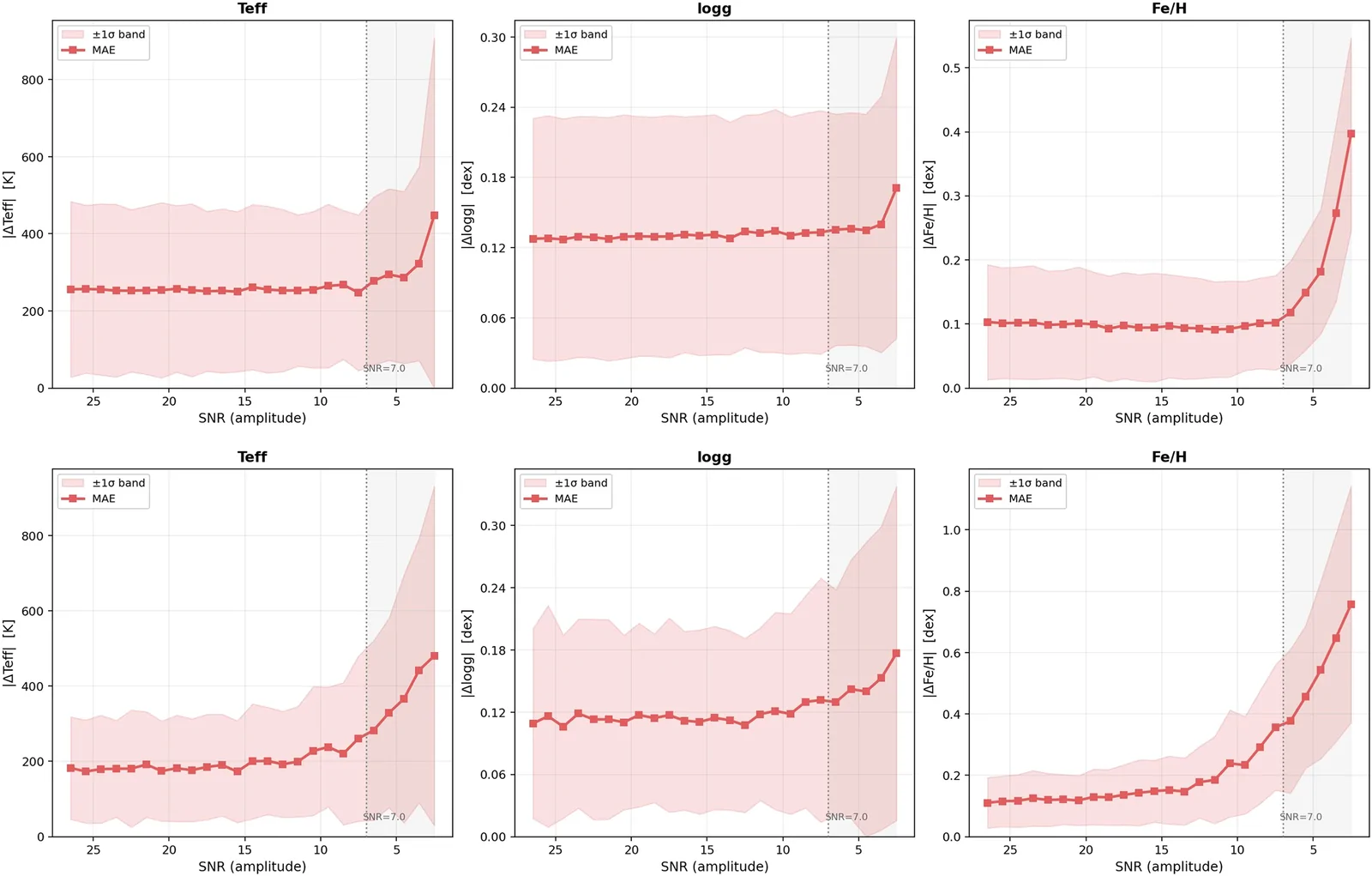
SNR-dependent prediction errors of the models for *T*_eff_ , log *g*, and [Fe∕H] The curves show the MAE and the corresponding ±1*σ* band as functions of the input SNR amplitude. During the generation process, we randomly selected 100 spectra from the high-SNR interval of 25–30 and progressively injected random noise at steps of 1 in SNR to construct controlled degradation tracks toward lower effective SNR levels. The upper row shows the resulting diagnostic after staged finetuning on the observational subsets with z-band SNR ∈ [9, 11] and SNR ∈ [7, 9], while the lower row shows the corresponding results before the staged low-SNR finetuning. Compared with the lower row, the upper row exhibits a smoother and more controlled error evolution as SNR decreases, indicating improved adaptation to the target low-SNR domain and consequently more stable stellar-parameter inference. The vertical dashed line marks SNR = 7.

This SNR-dependent behavior should be discussed against earlier LAMOST low-resolution studies. In the partially overlapping low-SNR regime, StarGRUNet reports uncertainties of 182 K in *T*_*eff*_, 0.34 dex in log *g*, and 0.07–0.17 dex for elemental abundances when *S*/*N*_*g*_∈[5, 10), whereas ESNet reports MAEs of 82 K, 0.20 dex, and 0.10 dex for *T*_*eff*_, log *g*, and [Fe/H], respectively, on its 0–10 low-SNR test set.^18^^,^^19^ By comparison, in Figure 6 the post-staged-finetuning checkpoint shows, over the overlapping SNR ≈5–10 interval, *T*_*eff*_ MAEs of roughly 250–300 K, log *g* MAEs of about 0.12–0.14 dex, and [Fe/H] MAEs of about 0.09–0.15 dex, while the pre-adaptation checkpoint is roughly 220–320 K, 0.12–0.15 dex, and 0.15–0.35 dex over the same interval. This means that, within the overlapping 5–10 regime, our log *g* prediction is clearly more accurate than both StarGRUNet and ESNet, our *T*_*eff*_ is broadly comparable with StarGRUNet but still worse than ESNet, while the improvement in [Fe/H] is less evident in this comparison. These differences are physically reasonable, because unlike the full-spectrum LAMOST settings adopted in the two earlier studies, our model is evaluated in a much more restrictive CaT-only 303-point setting and is explicitly optimized to survive transfer toward the pure ultra-low-SNR regime of [0, 3], rather than to maximize average accuracy over a broader low-SNR benchmark.

To quantify the robustness of the finetuned models across the full noise regime, we traced the mean absolute error and its 1*σ* dispersion for *T*_*eff*_, log *g*, and [Fe/H] as functions of the input SNR in Figure 6. In both checkpoints, the parameter errors remain relatively stable throughout the moderate-to-high SNR range and increase mainly when the input enters the ultra-low-SNR regime. Among the three labels, [Fe/H] is the most sensitive to decreasing SNR, whereas *T*_*eff*_ and log *g* degrade more gradually. After staged finetuning, the most identifiable gain appears in *l*og *g*, whose MAE over the overlapping SNR ≈5–10 interval remains at about 0.13–0.14 dex, compared with roughly 0.12–0.15 dex before adaptation. Over the same SNR interval, *T*_*eff*_ shows a modest improvement, with the error range changing from about 220 to 320 K before adaptation to about 250–300 K after adaptation. By contrast, [Fe/H] shows a clearer reduction in error in this comparison: its error range is about 0.10–0.18 dex after adaptation, compared with about 0.23–0.46 dex before adaptation. These results indicate that the effect of staged finetuning is label-dependent: it provides the clearest improvement for [Fe/H], noticeable stabilization for *T*_*eff*_, and more consistent behavior for log *g* over this SNR range. Therefore, the main value of staged finetuning lies not in uniformly reducing every error component; rather, although the degree of improvement varies across parameters, this remains acceptable given the higher sensitivity of metallicity estimation to noise.

### Time-related costs

For practical deployment, the computational cost of this pipeline remains moderate relative to the scale of current spectroscopic surveys. The pretraining stage was carried out on 4 × 80 GB NVIDIA H800 GPUs for about 2 × 24 h, corresponding to approximately 192 GPU-hours. Each finetuning stage required 4 × 80 GB H800 GPUs for about 1.2 × 24 h, i.e., about 115.2 GPU-hours per stage; under the present two-stage low-SNR adaptation, the total finetuning cost is therefore about 230.4 GPU-hours. The full training pipeline thus amounts to roughly 422.4 GPU-hours. At inference time, a single 16 GB RTX 4090 processes about 1,000 spectra per minute, implying that 10^6^ spectra can be evaluated in about 16.7 h on a single consumer-grade GPU. These figures suggest that, although staged adaptation introduces non-negligible additional training cost, the method remains operationally feasible for large-survey applications and can be scaled further through straightforward data-parallel inference.

### Data-related limitations

We addressed the resolution and noise-domain mismatch by continuously finetuning the full model on observational 303-wavelength spectra with a progressively lower-SNR curriculum, improving convergence and stellar-parameter inference.This staged adaptation is also important when viewed against previous LAMOST parameter-estimation studies. Earlier methods were typically assessed either on spectra with S/*N* ≥ 5 or on mixed low-SNR samples spanning 0–10, whereas our final target set is restricted to the more severe interval of SNR∈[0, 3]. Under such a stricter regime, some increase in parameter bias and dispersion is expected. The practical value of the present strategy is therefore not that it fully removes the low-SNR difficulty, but that it keeps the final predictions within a usable and physically interpretable range after transfer from synthetic 3,030-point spectra to observational 303-point spectra. This is especially important for [Fe/H], where the error grows most strongly as the spectra become more noise-dominated, yet the prediction still does not collapse into completely non-physical outputs.

**Figure 7:**
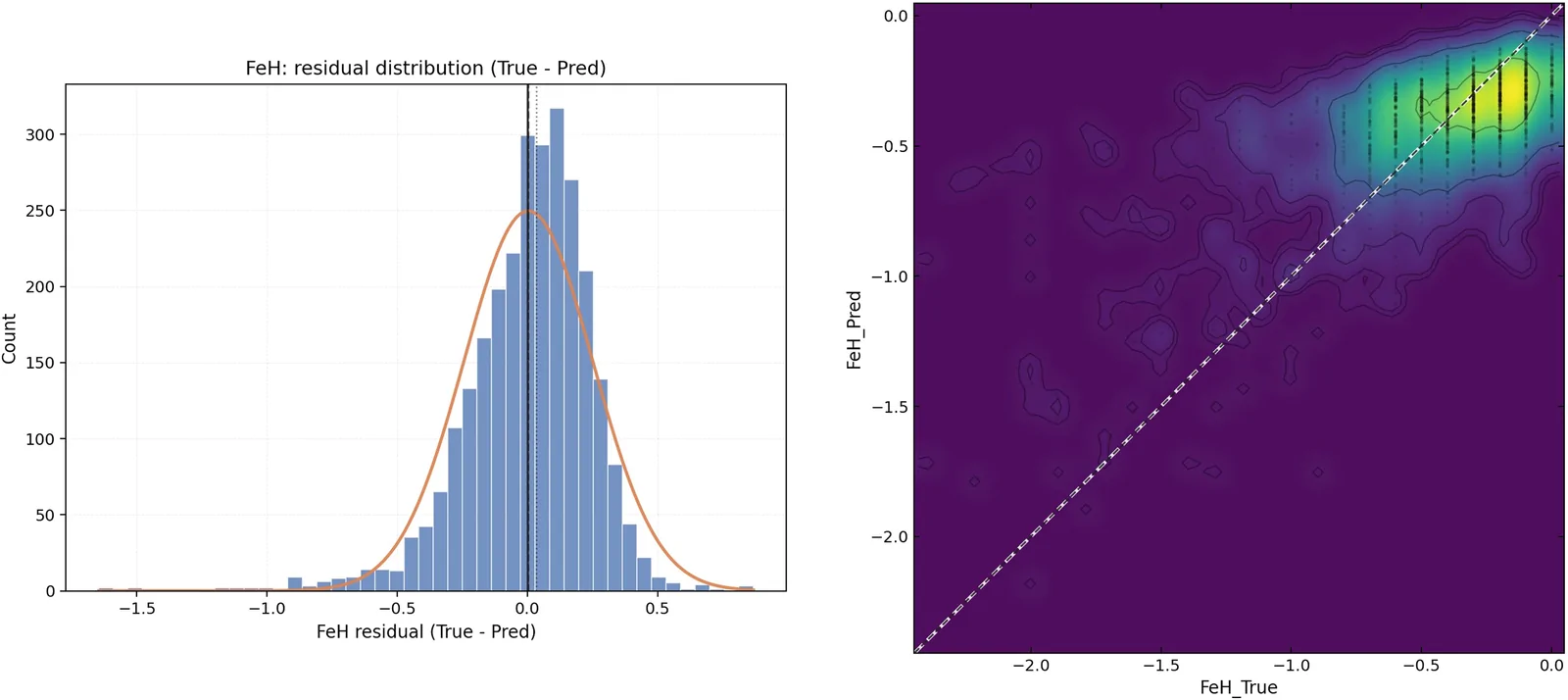
Additional diagnosis of metallicity inference on real observational 303-point spectra at approximately SNR ∼ 7 Left: distribution of metallicity residuals, defined as Δ[Fe∕H] = [Fe∕H]_true_ – [Fe∕H]_pred_. Right: density map of predicted versus true metallicity, where the dashed diagonal marks the one-to-one relation. The two images jointly show that the overall monotonic trend is preserved, but the predicted metallicity distribution is more concentrated than the true distribution, especially toward the metal-poor side.

As shown in Figure 7, the residual histogram and the [Fe/H]_*pred*_ – [Fe/H]_*true*_ density map provide a distribution-level diagnosis of metallicity inference on real observational 303-point spectra at approximately SNR ∼7. The dominant density ridge in the [Fe/H]_*pred*_ – [Fe/H]_*true*_ plane is still distributed roughly along the *y* = *x* diagonal, while the visible vertical banding along the [Fe/H]_*true*_ axis mainly reflects gaps in the LAMOST reference labels themselves. However, the overall agreement is clearly not ideal: the residual distribution is concentrated around ∼–0.5 rather than around zero, indicating a systematic offset in the bulk predictions. This behavior likely reflects, at least in part, a mismatch between the data prior learned in the early training stage and the label distribution of the real observational sample. At the same time, in the metal-poor regime below [Fe/H]<–1, the predictions do not collapse into an overly tight cluster, but instead remain somewhat spread with a slight bias toward lower values. This suggests that the initial pretraining has still captured a meaningful fraction of the metallicity-related structure, even though the recovery on real data remains biased.

This behavior is also qualitatively different from the way earlier LAMOST studies are usually summarized. StarGRUNet and ESNet mainly report average error metrics for low-resolution parameter inference,^18^^,^^19^ whereas Figure 7 in our setting highlights a distributional effect: the model preserves the overall metallicity ordering, yet the predicted metallicity distribution remains slightly more concentrated than the true one, implying a residual compression toward the central metallicity range. A plausible reason is the mismatch between the metallicity emphasis of the training prior and the label distribution of the real spectra. In our pretraining setup, the model is mainly exposed to metal-poor templates with [Fe/H]<–1, while the real spectra used in this diagnosis are more densely distributed in the interval −1≲[Fe/H]≲0. After the final observational finetuning, the prediction is therefore naturally pulled toward the denser central part of the real-data label distribution. Accordingly, at SNR∼7, the model preserves a physically meaningful metallicity ranking and retains useful object-level information, although the individual estimates remain coarse and the full spread of the metal-poor tail is still underestimated.

However, this bulk-level robustness does not remove several parameter-specific limitations at extremely low SNR. First, the inferred log *g* distribution remains relatively narrow (with a standard deviation of 0.095), even though the low-log *g* giant branch becomes visible in the Kiel diagram. While log *g* is the parameter that benefits most clearly from staged finetuning in our experiments, some degradation is still visible once the spectra enter the ultra-low-SNR regime. This likely reflects the reduced distinguishability of gravity-sensitive local spectral structure under severe noise contamination, together with a stronger influence of the learned prior when the observational evidence becomes weak. Even in that regime, however, the predictions remain positively correlated with the reference labels and are still informative at a coarse level.

Second, beyond the metallicity shrinkage discussed above, the [Fe/H] prediction also exhibits a clear restriction within the range [–2,0]. This limitation does not stem from an imbalance or incomplete coverage of extreme-metallicity objects in the training data, but rather from the absence of real spectral templates with [Fe/H]< –2 in our current training setup. Consequently, predictions in this regime are more vulnerable to a domain mismatch between the training distribution and the real observational sample, which may further affect the accuracy. Consequently, the current model should be regarded as a reasonably reliable metallicity estimator within the supported range of [–2, 0].

Finally, these limitations are likely exacerbated by observational systematics. The CaT lines lie in the red arm of the spectrum, adjacent to a dense forest of telluric water-vapor absorption features. Even when sky-line subtraction based on other fibers is applied, residual water-line contamination may persist at very low SNR, potentially altering the effective line profiles. This further degrades the usable information content for metallicity and gravity inference, forcing the model to rely more heavily on learned priors or to enter extrapolation-unstable regimes.

In addition, our synthetic degradation assumes a predominantly Gaussian noise model, whereas the noise in real low-SNR observations is likely a superposition of multiple components and is not fully described by a single Gaussian term. In particular, as SNR decreases, instrumental effects associated with the sky-background fibers and those associated with the target fibers may contribute differently, introducing extra systematics beyond the simplified simulation. As a result, the extracted line regions in real spectra often appear smoother and more weakly informative than the Gaussian-noised synthetic spectra, with both fine spectral information and apparent noise fluctuations being suppressed, which further increases the domain mismatch and can reduce the prediction accuracy.

**Figure 8:**
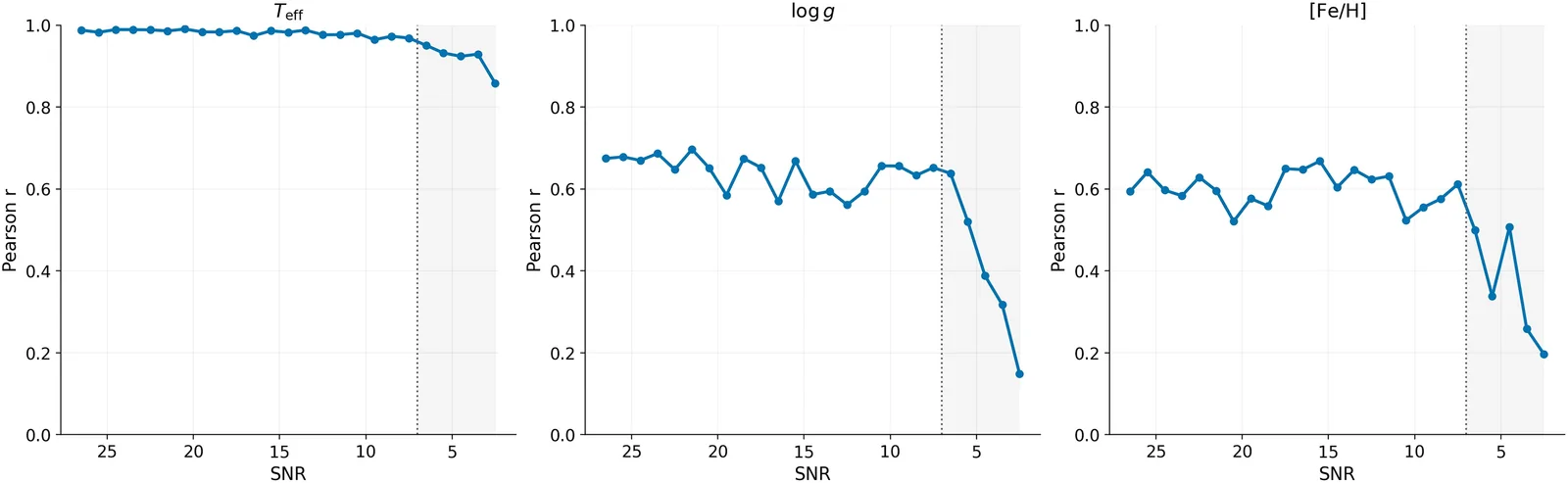
Prediction-label correlation as a function of SNR, evaluated on a random subset of 100 objects *T*_eff_ remains highly stable over most of the tested range, while log *g* and [Fe∕H] show a clearer SNR dependence in the lower-SNR regime. The vertical dashed line marks the transition near SNR ≈ 7, and the shaded region highlights the lower-SNR side.

**Figure 9:**
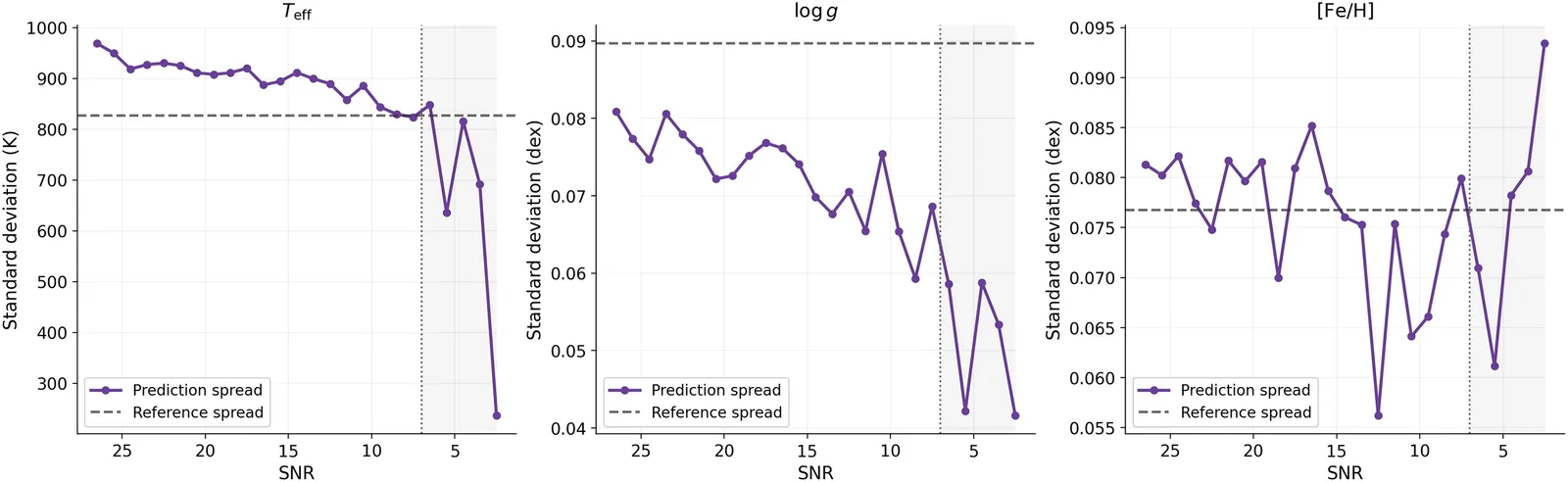
Across-object spread of predictions as a function of SNR, evaluated on a random subset of 100 objects The purple curve shows SD (pred) and the dashed line shows SD (true). *T*_eff_ remains comparatively stable, log *g* becomes more compressed at low SNR and [Fe∕H] remains informative.

The two SNR-diagnostic figures first make clear that degradation does occur as SNR decreases, and that the ultra-low-SNR regime is better interpreted as a progressively prior-dominated regime than as a complete collapse to a single prior value. As shown in Figure 8, *T*_*eff*_ remains the most robust parameter over most of the tested SNR range, with Pearson *r* staying high until the extreme low-SNR end. A plausible explanation is that the model has learned more continuum-level information during training, so temperature prediction can still rely on relatively global spectral-shape cues even after substantial degradation. By contrast, log *g* and [Fe/H] show more visible deterioration, which is consistent with a stronger domain shift for parameters that depend more directly on local line morphology. For log *g*, Figure 9 shows that the prediction spread becomes progressively compressed as SNR decreases, suggesting that under weakening observational evidence the model is increasingly driven toward a more regularized and conservative response, although a substantial positive correlation is still preserved over a broad SNR interval. For [Fe/H], the degradation is more pronounced for two related reasons: first, the metallicity-sensitive line information in the CaT region is progressively weakened as SNR decreases; second, for part of the sample, the local morphology in this wavelength range is itself less favorable for metallicity estimation, for example, when the spectra are dominated by Paschen-series features or when the CaT absorption is intrinsically shallow. Once SNR drops below approximately 7, the deterioration becomes markedly sharper, indicating that the error budget is no longer dominated mainly by idealized random noise. Instead, instrumental noise, sky-background-related noise, and data-reduction errors likely begin to play a leading role, and these effects are not fully captured by the Gaussian noise assumption adopted in our synthetic degradation. A similar behavior is also visible in Figure 9: both *T*_*eff*_ and log *g* exhibit noticeable degradation, while the [Fe/H] response becomes closer to a partly prior-driven estimate once the metallicity-sensitive signal is strongly suppressed. In this sense, these results suggest that the model still retains practically meaningful correlation in the low-SNR regime, but that its behavior becomes increasingly controlled by prior information and real-data domain shift as the observational evidence weakens.

### Future work

Our study was motivated by the need to extract useful information from low-SNR spectra that are often underused in large survey pipelines. While the present framework has demonstrated that this general paradigm is feasible, the discussion above also makes clear that several limitations of the current setup should be addressed before such a model can be used more broadly and more reliably. The next stage of this work will therefore focus on reducing the gap between synthetic training data and real observations, improving coverage in the currently underrepresented parts of parameter space, and making the method more robust under realistic survey conditions.

First, because the current degradation pipeline mainly assumes Gaussian noise, future work should introduce more realistic observational noise modeling that explicitly accounts for instrumental effects, sky-background subtraction, fiber-dependent systematics, and reduction-induced errors, especially in the regime of SNR≲7.

Second, because the present real spectral templates training set does not cover the EMP regime below [Fe/H]< –2 and does not fully represent the diversity of CaT-region morphologies, future work should extend the template grid and improve coverage of low-SNR real spectra with reliable external labels.

Third, because the observational reference labels are not fully continuous and may themselves contain gaps or ambiguity, future work should incorporate uncertainty-aware supervision or probabilistic calibration rather than treating all labels as equally precise point targets.

Finally, because the current framework has so far been validated mainly in a controlled proof-of-concept setting, future work should test its stability, uncertainty calibration, and out-of-distribution behavior across realistic survey conditions, instruments, and reduction pipelines.

### Limitations of the study

In results, we find that a model pretrained and finetuned on simulated 3,030-wavelength spectra performs poorly on observational 303-wavelength spectra, initially attributed to SNR ≤ 3 and potential distortion from interpolating sparse spectra. Finetuning on a mix of simulated and observational low-SNR 303-point data yielded limited gains, as simulated noise exhibits high-frequency oscillations unlike the smoother observational spectra, biasing the model toward spurious features. To address this, we adopted continuous full-parameter finetuning solely on observational 303-point spectra, using a staged curriculum that progressively lowers SNR. This strategy enables effective adaptation to the combined resolution and noise shift, leading to rapid convergence and improved stellar-parameter inference from low-SNR data.

## Resource availability

### Lead contact

Further information and requests for resources should be directed to and will be fulfilled by the lead contact, Cunshi Wang (wangcunshi@nao.cas.cn).

### Materials availability

This study did not generate new unique materials.

### Data and code availability

• The raw synthetic spectra used in this study are publicly available from the PHOENIX Göttingen spectral library. The observational spectra and stellar-parameter labels are publicly available from LAMOST Data Release 12 low-resolution spectra.
• The complete code base, data-processing scripts, and model resources for this study are available at: https://github.com/Yu-Yang-Li/StarWhisper/tree/main/Low-SNR-Stellar-Spectra-as-Language.
• Any additional information required to reproduce or reanalyze the results reported in this paper is available from the lead contact upon reasonable request.

## Acknowledgments

The support came from the Science and Education Integration Funding of 10.13039/501100011332University of Chinese Academy of Sciences. We also received backing from the Strategic Priority Program of the 10.13039/501100002367Chinese Academy of Sciences under grants XDB41000000, XDB0550000, XDB0550100, and XDB0550102. Additional support came from the 10.13039/501100001809National Natural Science Foundation of China (NSFC) under grants NSFC-11988101, 11973054, and 12090040. Special acknowledgment goes to the China Manned Space Project for their science research grant, denoted by NO.CMS-CSST-2021-B07. The research is also supported by 10.13039/501100015060National Astronomical Observatories, Chinese Academy of Sciences no. E4TG2001. J.L. extends gratitude for the support received from the New Cornerstone Science Foundation, particularly via the New Cornerstone Investigator Program, and the honor of the XPLORER PRIZE. Guoshoujing Telescope (the Large Sky Area Multi-Object Fiber Spectroscopic Telescope [LAMOST]) is a National Major Scientific Project built by the 10.13039/501100002367Chinese Academy of Sciences. Funding for the project has been provided by the 10.13039/501100010453National Development and Reform Commission. LAMOST is operated and managed by the National Astronomical Observatories, Chinese Academy of Sciences.

## Author contributions

Conceptualization, Y.L., C.W., and A.L.; methodology, J.X., X.S., and Y.L.; software, J.X., X.S., Y.L., C.W., and Z.F.; validation, J.X., X.S., Y.L., and C.W.; formal analysis, J.X., X.S., Y.L., and C.W.; investigation, Y.L. and C.W.; resources, A.L. and J.L.; data curation, C.W. and Z.F.; writing – original draft, J.X. and X.S.; writing – review and editing, J.X., X.S., Y.L., and C.W.; visualization, J.X. and X.S.; supervision, A.L. and J.L.; project administration, Y.L, C.W., and A.L.; funding acquisition, A.L. and J.L.

## Declaration of interests

The authors declare that they have no known competing financial interests or personal relationships that could have appeared to influence the work reported in this paper.

## STAR★Methods

### Key resources table

**Table:** 

REAGENT or RESOURCE	SOURCE	IDENTIFIER
**Deposited data**		

PHOENIX synthetic spectra/PHOENIX model-atmosphere grid	PHOENIX stellar atmosphere library; Husser et al.	PHOENIX Göttingen spectral library; PHOENIX models described by Husser et al., 2013, https://doi.org/10.1051/0004-6361/201219058
PHOENIX-derived synthetic spectra used for pretraining	This paper; derived from PHOENIX synthetic spectra	Generated from PHOENIX spectra in the Ca II triplet region; wavelength window: 8,450–8,710 Å; degraded to *R* = 1,800; generation scripts available in the code repository
LAMOST DR12 low-resolution spectra	LAMOST DR12 LRS	LAMOST Data Release 12 v0; low-resolution spectra and catalog access through the LAMOST DR12 data portal
LAMOST DR12-derived observational subsets	This paper; derived from LAMOST DR12 LRS	Derived subsets used for staged finetuning and inference; includes SNR-z ∈[9,11], SNR-z ∈[7,9], and SNR-z ≤3 subsets; selection and processing scripts available in the code repository
Stellar-parameter labels	LAMOST DR12 stellar-parameter catalog	*T*_*eff*_, *logg*, and [Fe/H] labels used for supervised finetuning and evaluation

**Software and algorithms**		

Code repository	This paper	https://github.com/Yu-Yang-Li/StarWhisper/tree/main/Low-SNR-Stellar-Spectra-as-Language
Digit-wise decimal spectral tokenizer	This paper	Available in the code repository
Order-agnostic spectral Transformer/masked-diffusion backbone	This paper	Available in the code repository; approximately 130M parameters
PHOENIX preprocessing pipeline	This paper	Available in the code repository; includes parameter interpolation, CaT-window extraction, resolution degradation, and synthetic-noise injection
Self-supervised pretraining pipeline	This paper	Available in the code repository
SNR-graded finetuning and regression pipeline	This paper	Available in the code repository
Evaluation pipeline	This paper	Available in the code repository; includes RMSE, MAE, SNR-binned diagnostics, and population-level Kiel-diagram analysis
Software environment	This paper	Python, PyTorch, NumPy, SciPy, scikit-learn, and XGBoost; exact package versions are provided in the code repository
Pretrained spectral backbone checkpoint	This paper	Available through the code repository
Final low-SNR finetuned checkpoint	This paper	Available through the code repository

**Others**		

Computing hardware	This paper	× NVIDIA H800 80 GB GPUs for pretraining and finetuning; NVIDIA GeForce RTX 4090 GPU for inference-speed evaluation

### Experimental model and study participant details

This study did not involve human participants, animals, cell lines, or biological samples. The analysis was based on publicly available astronomical spectra and synthetic stellar-atmosphere spectra.

#### Data

##### PHOENIX

The PHOENIX spectral model^20^^,^^21^ is a state-of-the-art stellar atmosphere code that generates high-resolution synthetic spectra across UV to IR wavelengths. Assuming local thermodynamic equilibrium, it self-consistently solves radiative transfer and hydrostatic equilibrium, incorporating atomic and molecular opacities, dust formation in cool stars, and mixing-length convection. Spectra are computed as a function of *T*_*eff*_, *logg*, and [Fe/H], and are widely used for spectral classification, stellar parameter determination, and exoplanet host star modeling—serving as a key benchmark in modern astrophysics. In our work, the PHOENIX also refers to the synthetic spectra generated by the PHOENIX stellar atmosphere model.

Since we aim to search low-metallicity stars by training the model, we select the spectra in the parameter space *T*_*eff*_ in [2,300,12,000] K, *logg* in [0,6] dex, [*Fe*/H] in [–4.0, –1.0] dex. In PHOENIX, the parameter gap follows 100 K for *T*_*eff*_≤7,000 K otherwise 200 K gap, 0.5 dex for *logg*, and 1.0 dex for [Fe/H]. The gap is too large for adopting these parameters as a training template, so we densify these gaps into 1/10 of the original ones, and this will result in 10 K for *T*_*eff*_≤7,000 K otherwise 20 K gap, 0.05 dex for *logg*, and 0.1 dex for [Fe/H].

##### Pre-processing

In preprocessing, we generate a template sample set through three steps: (1) interpolating PHOENIX spectra to reduce stellar parameter gaps and expand the training set; (2) selecting a wavelength region representative of low-metallicity stars and degrading spectral resolution to R = 1,800 to match LAMOST low-resolution spectra (LRS); and (3) adding noise to simulate realistic observational spectra.

*Interpolation of spectra*. To generate a comprehensive template set with a sufficiently dense parameter distribution for model training, we interpolate the PHOENIX spectra following the method described in Xiang et al.^9^ This process involves performing linear interpolation using the eight nearest parameter grid points surrounding a target (*T*_*eff*_, *logg*, [Fe/H]). The detailed mathematical formulation and the coordinate definitions for this trilinear interpolation scheme are presented in supplemental information. After doing the parameter interpolation, we get more than 2,000,000 templates.

*Sampling and adding noise*. We select the spectral window from 8,450 Å to 8,710 Å of the original PHOENIX spectra (over 20,000 flux points) to reduce high dimensionality and focus on physically diagnostic features. This region encompasses the Ca II triplet (CaT), a key tracer for stellar metallicity.

We apply Gaussian convolution to match the resolution of PHOENIX spectra (*R* = 10,000) to that of LAMOST LRS (*R* = 1,800). Observational noise is approximated as additive white Gaussian noise to capture dominant stochastic fluctuations in localized spectral regions. Detailed information on the convolution width and noise modeling is provided in supplemental information.

A small amount of noise is injected into the high-SNR data (SNR >100) to construct a high-SNR synthetic dataset for pretraining, thereby establishing robust feature representations. During model evaluation, we progressively reduce the SNR by adding noise to assess the model under increasingly noisy observational conditions.

After preprocessing, we construct a high-SNR dataset covering the CaT region, populated with synthetic spectra sampled across a dense grid of stellar parameters.

##### LAMOST LRS

We adopt LAMOST Data Release 12 (DR12) LRS, covering 3,700–9,000 Å, as our target dataset. To support staged curriculum fine-tuning (finetune), we construct two metal-poor ([Fe/H] ≤0) observational subsets, for each containing about 100,000 spectra, with z-band SNR in [9, 11] and [7, 9], respectively.

Additional subsets include high-SNR spectra for initial transfer learning and an ultra-low-SNR set (SNR ≤3) for final prediction. All selections prioritize z-band SNR (SNR-z) due to its strong correlation with the CaT region used in training.

The sample is dominated by F–K main-sequence stars, with sparse coverage at high *T*_*eff*_, low *logg*, or low [*Fe*/H]. To better represent these rare populations, we augment the sample by generating two stars in the region *T*_*eff*_∈[8,000 K,9,000 K], *logg*∈[2,3], and [*Fe*/H]∈[-1.5,-2] , and three more for *T*_*eff*_>9,000 K, *logg* < 2, and [*Fe*/H]<-2.

Each augmentation rule is applied independently; stars meeting multiple criteria are augmented once per rule, cumulatively increasing the synthetic sample size. Augmentation adds Gaussian noise to observed spectra (sampling and adding noise), with SNR randomly drawn from 2.5 to 3.0 for each instance, followed by wavelength trimming to match the pretraining simulated dataset.

Compared to the original, augmentation increases sample density in previously underrepresented regions, yielding a more balanced training set. A conservative strategy is adopted to prevent over-representation of rare types from limited inputs, ensuring physical plausibility.

### Method details

This section briefly outlines the implementation process and approach of our work.

We pretrain the backbone via *spectral prediction*: given a partially observed spectrum, predict its masked part, an objective analogous to masked modeling that yields transferable representations in language and vision.

We first implement this pretraining and an initial finetuning stage on theoretical data, and then transfer the model to observational data through full-parameter finetuning. To mitigate the distribution discrepancy and avoid exposing the model too abruptly to the most challenging, very low- *SNR* spectra, we begin this transfer stage by finetuning on a high- *SNR* subset, and then carry out several successive rounds of full finetuning on datasets with progressively lower *SNR*, effectively implementing a staged curriculum in *SNR*. Subsequently, we introduce spectral discretisation and token construction, present the masked spectral pretraining method, describe the downstream regression finetuning and evaluation proposal, and provide a universal paradigm for the general representation and application of spectra.

#### Tokenization

##### Digit-wise decimal quantization and decomposition

This subsection describes how astronomical spectra and their physical labels are converted into symbolic sequences suitable for language-model training. The central motivation is to avoid representing each continuous scalar value as a single large discrete class. A conventional large-bin discretization would require the model to distinguish among thousands or even tens of thousands of possible bins for each value, which substantially increases the difficulty of learning. In contrast, digit-wise decimal quantization decomposes each scalar into a short sequence of decimal digits with explicit position tags. This keeps the vocabulary compact while preserving numerical precision at the level required for spectral modeling.

We use a unified token grammar for both flux values and physical labels. Each digit token consists of a prefix indicating the quantity type, a decimal digit from 0 to 9, and a position suffix. The prefix S denotes spectral flux, while T, L, and F denote *T*_*eff*_, *logg*, and [*Fe*/H], respectively. The position suffixes include _tthou, _thu, _hun, _ten, and _one, corresponding to the ten-thousand, thousand, hundred, ten, and one positions. The vocabulary also includes three special tokens: <BOS> for the beginning of a sequence, <EOS> for the end of a sequence, and <SEP> for padding.

For the spectral sequence, each continuous flux value is first mapped to a four-digit decimal code and then represented by four consecutive flux tokens of the form S<digit>_<pos>. These four tokens correspond to the thousand, hundred, ten, and one positions of the quantized flux value. For example, a flux code with digits (7,4,4,0) is represented as S7_thu, S4_hun, S4_ten, and S0_one. Therefore, a spectrum with *N* wavelength positions is transformed into an ordered sequence of 4*N* flux tokens, with <BOS> and <EOS> marking the sequence boundaries and <SEP> used when padding is required. This representation converts the original continuous spectral input into a language-model-compatible token sequence while retaining an approximately invertible mapping back to flux space.

Physical labels are encoded using the same digit-wise principle. Specifically, *T*_*eff*_ is represented by five digit tokens with the T prefix, including the ten-thousand position. The surface gravity *logg* is represented by a sign token, either L_pos or L_neg, together with three digit tokens at the hundred, ten, and one positions. The metallicity [*Fe*/H] is represented by a sign token, either F_pos or F_neg, together with two digit tokens at the ten and one positions. In the current work, pretraining is mainly performed on spectral S tokens, while the physical-label tokens T, L, and F are introduced in finetuning so that stellar-parameter regression can be formulated as sequence generation.

The detailed numerical mappings, including clipping, quantization, digit extraction, and inverse decoding, are provided in supplemental information.

#### Model architecture

**In this work, we develop a self-supervised masked-diffusion model for digit-tokenized spectra and use the pretrained backbone for downstream prediction of physical parameters.** Through pretraining and finetuning, the model targets feature extraction under low- *SNR* conditions, enabling accurate parameter inference and efficient candidate selection.

As shown in Figure 1, the model consists of an embedding base, a conditioned Transformer backbone, and task-specific decoders. The overall architecture is designed to learn contextual spectral representations for masked reconstruction in pretraining and to transfer them to stellar-parameter regression in finetuning. Detailed descriptions are given in model architecture.

##### Data processing workflow

*Input and discretisation*. This step treats the position as the minimal physical unit (each position corresponds to four tokens), converts the continuous flux stream into stable and reversible digit symbols, and turns spectral modeling into a fixed-vocabulary multi-class problem.

*Structured arbitrary-order sampling*. For each position, define a four-token position group $g_{j}=\left(d_{j}^{\left(10^{3}\right)},d_{j}^{\left(10^{2}\right)},d_{j}^{\left(10^{1}\right)},d_{j}^{\left(10^{0}\right)}\right)$ for *j* = 1, …,*N*. Let $S_{N}$ be the symmetric group on *N* indices. A sample $\sigma \in S_{N}$ permutes only the position groups while preserving the fixed order within each group. Lifting *σ* to token indices gives *π* = *lift*(*σ*), which changes the group order but keeps the four-digit order within each position unchanged. Training follows the curriculum Random_CL: with probability *p*_*rand*_ the permuted order *π* is used, otherwise the standard left-to-right autoregressive order is used. validation and inference always use the autoregressive order. This strategy preserves position atomicity and strengthens the model’s ability to perform imputation and denoising at arbitrary spectral locations, which better reflects observational spectra with discrepancy or low *SNR* segments than a strictly left-to-right scheme.

*Embedding encoder and conditional injection*. For the token sequence ***y***_*π*_(1:*T*), we use three embeddings and one conditioning channel.

wte (token embedding) is implemented as *E*_*tok*_∈R^*V*×*d*^, where *V* is the vocabulary size and *d* = 256; it maps each discrete token into a *d*-dimensional vector. The four digit tokens belonging to the same position are embedded separately but always appear in the fixed intra-position order (thousand, hundred, ten, one), so that the position remains the minimal physical unit.

wpe (absolute position embedding) is implemented as *E*_*pos*_∈R^*T*×*d*^ and encodes the absolute wavelength-bin index of each token (*T* is the sequence length). This allows the model to distinguish where in the spectrum a local pattern occurs and to relate tokens across distant wavelength segments.

wtpe (target-position embedding) is implemented as *E*_*tpos*_∈R^*T*×*c*^ with *c* = *cond*_*dim* = 128. Unlike wte and wpe, it is not added to the token features; instead, its value at the current prediction position serves as the conditioning signal *C* that is fed to every layer. Through AdaLN, this conditioning produces per-layer shift, scale, and gating parameters that modulate both the self-attention and the MLP blocks in a target-aware manner.

From a physical perspective, wte and wpe together enable the network to recognize line cores, wings, and continuum regions at specific wavelengths, while wtpe explicitly specifies the position being inferred and biases the model toward aggregating spectrally relevant evidence for that target position.

*Conditioned transformer propagation*. The backbone is formed by stacked conditioned Transformer blocks, which model both local spectral features and long-range contextual dependencies under the guidance of the target-position condition, thereby producing spectral representations relevant to the current prediction position.

*Final layer and decoders*. After the final conditioning stage, the hidden representation is passed to task-specific output heads: a token decoder for discrete flux reconstruction during pretraining, and a regression head for stellar-parameter prediction during finetuning. Further details of the architecture and decoding procedure are given in model architecture.

##### Loss function

We use focal loss for token-level spectral reconstruction,^22^ because digit-token frequencies are imbalanced and many positions correspond to easy continuum tokens. Focal modulation down-weights these easy tokens and emphasizes rare or noise-sensitive spectral tokens, which are more informative for learning spectral structures. The downstream stellar-parameter task is optimized separately with MSE in the standardized continuous label space.

#### Pretraining

##### Pretraining data and order-agnostic spectral reconstruction

From a data perspective, high- *SNR* synthetic spectra are used during the initial pretraining phase. This design enables the model to first learn fine-grained spectral line structures at the position level and develop robust flux prediction capabilities, thereby establishing a solid foundation for subsequent incremental pretraining on observational data and finetuning for physical parameter regression.

In methodology, we adopt an order-agnostic masked generation view for discrete token sequences.^23^ With the above design, the model can effectively learn position-level spectral line structures and their contextual correlations during pretraining. Per-digit predictions are reassembled into per-position flux, and *Flux RMSE* is computed accordingly, so that the symbolic objective remains consistent with the diagnostic of position physical quantities during pretraining.

#### Optimization and learning-rate schedule

We adopt AdamW together with a warmup plus cosine-annealing learning-rate schedule, which stabilizes long-sequence training and yields more consistent reconstruction of spectral-line details while reducing noise fitting. The token-level objective uses focal loss to alleviate class imbalance and easy-example dominance, thereby improving robustness and sample efficiency in low- *SNR* scenarios. The pretrained representation is directly reused in the finetuning stage: mask-aware mean aggregation is applied to valid tokens, and a lightweight regression head is attached to map the “spectral representation that can be completed” with relatively minor changes to supervised regression of *T*_*eff*_, *logg*, and [*Fe*/H].

#### Finetune

**This section primarily outlines the workflow for finetuning and regression head design based on pretrained models**. It addresses the challenge of effectively utilizing low- *SNR* data mentioned earlier, resolving the task of performing physical parameter regression efficiently under such conditions.

We keep the same discrete input sequence as in pretraining, remove structure-only special tokens, and apply masked mean pooling over valid tokens to obtain a sequence-level representation. The pooled representation is fed into a lightweight two-layer regression head to predict the three physical parameters. To reduce scale-induced imbalance across targets, we compute per-parameter means and standard deviations on the training set and train the regressor by minimizing mean squared error in the standardized space; at inference, outputs are de-standardized back to physical units.

After pretraining and finetuning on 3,030-position synthetic spectra, the model exhibits strong performance in the simulated setting; however, a direct transfer to 303-position observational spectra does not meet the desired accuracy. To bridge this gap, we perform successive rounds of full-parameter finetuning on 303-position observational spectra with gradually decreasing *SNR*, starting from a high- *SNR* subset with *SNR* > 100 (one million spectra) and then extending to lower- *SNR* regimes, specifically *SNR* in [9,11], and *SNR* in [7,9]. During this staged curriculum, the model converges rapidly and achieves competitive performance across *SNR* levels. **This behavior demonstrates that the pretrained backbone can effectively learn line features embedded in low-***SNR* **spectra and regress the physical parameters, successfully completing the transfer from high-***SNR* **synthetic spectra to low-***SNR* **observational spectra and making practical use of low-***SNR* **astronomical data.**
