# Supplementary material for: Astronomical spectra as language: Order-agnostic foundation model for low-SNR reconstruction and stellar parameter prediction
Source: iScience. 2026 Aug 22;29(9):117165. doi: 10.1016/j.isci.2026.117165 (PMC13524736; doi:10.1016/j.isci.2026.117165)
Supplement: Supplementary file 1 [file mmc1.pdf]

iScience, Volume *29*

Supplemental information

Astronomical spectra as language:

Order-agnostic foundation model for low-SNR

reconstruction and stellar parameter prediction

Jiacheng Xu, Xinrui Song, Yuyang Li, Cunshi Wang, Zhiwen Fu, Ali Luo, and Jifeng Liu

A. Details of Spectral Interpolation Method

In this supplementary material, we detail the interpolation scheme mentioned in the main text. Define the flux of the spectrum at wavelength λ, with stellar parameters (T, G, F) corresponding to $T_{\rm eff}$, $\log g$, and [Fe/H], as $f(T, G, F, \lambda)$. The target parameters lie within the bounds:

$$T_1 \le T \le T_2, \quad G_1 \le G \le G_2, \quad F_1 \le F \le F_2.$$

Here, the T_is, G_is, and F_is represent the nearest grid points of stellar parameters in the PHOENIX spectra. Assuming a non-degenerate problem (i.e., not situated strictly on the boundary), we define the normalized distance variables:

$$t = \frac{T - T_1}{T_2 - T_1}, \tag{1}$$

$$g = \frac{G - G_1}{G_2 - G_1}, \tag{2}$$

$$m = \frac{F - F_1}{F_2 - F_1}. \tag{3}$$

The fluxes at the eight corners of the parameter cube are denoted as:

$$\begin{aligned}
f_{111} &= f(T_1, G_1, F_1, \lambda), \quad & f_{112} &= f(T_1, G_1, F_2, \lambda),\\
f_{121} &= f(T_1, G_2, F_1, \lambda), \quad & f_{122} &= f(T_1, G_2, F_2, \lambda),\\
f_{211} &= f(T_2, G_1, F_1, \lambda), \quad & f_{212} &= f(T_2, G_1, F_2, \lambda),\\
f_{221} &= f(T_2, G_2, F_1, \lambda), \quad & f_{222} &= f(T_2, G_2, F_2, \lambda).
\end{aligned}$$

The final interpolated spectrum $f(T, G, F, \lambda)$ is calculated as:

$$\begin{aligned}
f(T, G, F, \lambda) = &(1-t)(1-g)(1-m) \cdot f_{111} + (1-t)(1-g)m \cdot f_{112}\\
&+ (1-t)g(1-m) \cdot f_{121} + (1-t)gm \cdot f_{122}\\
&+ t(1-g)(1-m) \cdot f_{211} + t(1-g)m \cdot f_{212}\\
&+ tg(1-m) \cdot f_{221} + tgm \cdot f_{222}.
\end{aligned} \tag{4}$$

B. Details on Feature Selection and Noise Modeling

This supplementary material provides the physical motivation for feature selection, the mathematical details of resolution broadening, and a discussion on noise characteristics.

Among diagnostic spectral features, the CaT, comprising lines at approximately 8,498 Å, 8,542 Å, and 8,662 Å, plays a critical role. These lines are highly sensitive to stellar metallicity and remain strong in the near-infrared region where atmospheric absorption is relatively low. This makes the CaT particularly effective for detecting metal-poor stars, even in heavily reddened regions of the Galaxy. Observations centered on this feature have substantially advanced our understanding of the distribution and properties of low-metallicity stars[1,3].

To simulate the decrease in resolution from PHOENIX ($R_{\rm high} = 10,000$) to LAMOST LRS ($R_{\rm low} = 1,800$), we apply a Gaussian convolution. The standard deviation σ_G is given by:

$$\sigma_G = \frac{\lambda_{\rm FWHM}}{2.355} \sqrt{\frac{1}{R_{\rm low}^2} - \frac{1}{R_{\rm high}^2}}, \tag{5}$$

where $\lambda_{\rm FWHM}$ is taken as 8,580 Å, representing the central wavelength of the spectral region of interest.

In low-SNR astronomical spectra, distinct noise patterns are commonly observed, including photon noise, readout noise, and sky background fluctuations[4]. Poisson noise becomes significant in regions with low photon counts, leading to asymmetric distributions. Additionally, instrumental instabilities and calibration errors can introduce red noise, a form of correlated, low-frequency noise that mimics broad spectral features[2]. Although these components are present in observations, the dominant noise in localized spectral analyzes can be effectively approximated as white noise. Therefore, we model the noise within the CaT region as additive white Gaussian noise, simplifying characterization while retaining essential statistical properties.

C. Tokenization

C.1. Digit-wise Decimal Quantization and Decomposition: Implementation Details

This supplementary subsection records the concrete numerical mappings used in our digit-wise discretisation. The motivation, background and basic token grammar (prefixes `S/T/L/F`, position tags, and examples such as `S7_thu`) have already been described in the Tokenization section of the main text, so we do not repeat them here.

C.2. Flux Discretisation

Let a continuum-normalized spectrum be $f_{1:N}$, and denote the n -th normalized spectral flux (at a wavelength position) by $f_n \in [0, 1]$. We first clip values to a fixed range,

$$\tilde{f}_n \;=\; \mathrm{clip}(f_n, 0, 1), \tag{6}$$

and then quantize onto a four-digit decimal grid,

$$q_n \;=\; \mathrm{clip}\big(\lfloor 9999\, \tilde{f}_n \rfloor, 0, 9999 \big) \in \{0, \ldots, 9999\}. \tag{7}$$

Equivalently, the quantized value can be written as

$$q_n \;=\; 1000\, d_n^{(10^3)} + 100\, d_n^{(10^2)} + 10\, d_n^{(10)} + d_n^{(1)}, \qquad d_n^{(10^k)} \in \{0, \ldots, 9\}, \tag{8}$$

so that each position is represented by its four decimal digits.

For implementation, we define the digit-position set $\mathcal{P} = \{\mathrm{thu}, \mathrm{hun}, \mathrm{ten}, \mathrm{one}\}$ with

$$\pi(\mathrm{thu}){=}3, \quad \pi(\mathrm{hun}){=}2, \quad \pi(\mathrm{ten}){=}1, \quad \pi(\mathrm{one}){=}0. \tag{9}$$

The digit at position $\ell \in \mathcal{P}$ is then

$$d_n^{(\ell)} \;=\; \left\lfloor \frac{q_n}{10^{\pi(\ell)}} \right\rfloor \bmod 10, \qquad \ell \in \mathcal{P}, \tag{10}$$

and is mapped to the corresponding flux token (e.g. `S7_thu` , `S4_hun` , etc.) as described in the main text. In the reverse direction, we can approximately recover the normalized flux via

$$\tilde{f}_n \;\approx\; \frac{q_n}{9999}, \tag{11}$$

which allows position-level RMSE to be monitored consistently during pretraining.

C.3. Physical-parameter Discretisation

For the physical labels, we use the same digit-wise idea but with different scales and digit counts.

We first define the integer grids

$$z_{\mathrm{teff}} = \mathrm{round}(T_{\mathrm{eff}}), \quad z_{\log g} = \mathrm{round}(100\,|\log g|) \in \{0, \ldots, 999\}, \quad z_{\mathrm{FeH}} = \mathrm{round}(10\,|[\mathrm{Fe/H}]|) \in \{0, \ldots, 99\}. \tag{12}$$

Each integer is then decomposed into decimal digits

$$z = \sum_k d^{(10^k)}\, 10^k, \qquad d^{(10^k)} \in \{0, \ldots, 9\}, \tag{13}$$

and mapped to position-specific tokens: T_{eff} uses five digits (ten-thousand, thousand, hundred, ten, one) ; $\log g$ uses three digits (hundred, ten, one) plus a sign token L_{pos} or L_{neg} ; [Fe/H] uses two digits (ten, one) plus a sign token F_{pos} or F_{neg} . The exact token forms (e.g. `T0_tthou`, `L3_hun`, `F7_one`) and the total vocabulary size are already given in the main text.

At inference and evaluation time, we map the discrete outputs back to the physical domain via

$$\widehat{\log g} = s_{\log g}\, \frac{z_{\log g}}{100}, \qquad \widehat{[\mathrm{Fe/H}]} = s_{\mathrm{FeH}}\, \frac{z_{\mathrm{FeH}}}{10}, \tag{14}$$

where $s_{\log g}, s_{\mathrm{FeH}} \in \{+1, -1\}$ are determined by the sign tokens (`L_pos`, `L_neg`, `F_pos`, `F_neg`) . For numerical consistency, the signed-zero case (-0.0) is canonicalized to 0.0.

In practice, the pre-processing pipeline simply applies these formulas to convert observationally valued fluxes and labels into digit-wise tokens, and the inverse formulas are used to decode model outputs back to spectra and physical parameters.

D. Model Architecture and Training

D.1. Input Sequence and Arbitrary-order Sampling

After preprocessing and digit-wise discretisation, each spectrum is grouped by `spectrum_id` and sorted by wavelength-position index. Each position contributes four ordered digit tokens,

$$g_j = \left(d_j^{(10^3)},\ d_j^{(10^2)},\ d_j^{(10^1)},\ d_j^{(10^0)}\right), \qquad j = 1, \ldots, N,$$

where N is the number of wavelength positions in the spectrum. The resulting token sequence is

$$\mathbf{y} = \left[\texttt{<BOS>},\ d_1^{(10^3)},\ d_1^{(10^2)},\ d_1^{(10^1)},\ d_1^{(10^0)},\ \ldots,\ d_N^{(10^3)},\ d_N^{(10^2)},\ d_N^{(10^1)},\ d_N^{(10^0)},\ \texttt{<EOS>},\ \texttt{<SEP>},\ \ldots\right].$$

For arbitrary-order training, we permute the position groups while keeping the within-position digit order fixed. Let $\sigma \in \mathcal{S}_N$ be a permutation over the N positions. The lifted permutation acts on the grouped sequence as

$$\pi = \mathrm{lift}(\sigma) : [g_1, g_2, \ldots, g_N] \longmapsto [g_{\sigma(1)}, g_{\sigma(2)}, \cdots, g_{\sigma(N)}].$$

The training curriculum uses `Random_CL`: with probability p_{rand} the permuted order π is used, and otherwise the canonical autoregressive order is used. Validation and inference always use the autoregressive order. This construction changes only the order of position groups and preserves the local four-digit structure of each wavelength position.

D.2. Embedding Encoder and Conditioning

Given a sampled order $\mathbf{y}_\pi(1\!:\!T)$, we construct the initial hidden states from token embeddings and absolute position embeddings. Specifically,

$$h^{(0)} = \left[\, E_{\mathrm{tok}}\left(\mathbf{y}_\pi(1\!:\!T)\right) \right] + \left[\, E_{\mathrm{pos}}(0)\,;\ E_{\mathrm{pos}}\left(\pi(1\!:\!T)\right) \right] \in \mathbb{R}^{B\times T\times d},$$

where E_{tok} denotes the token embedding table, E_{pos} denotes the absolute position embedding, B is the batch size, T is the padded sequence length, and d is the hidden dimension.

In parallel, we construct a target-position conditioning channel,

$$C = \left[\, E_{\mathrm{tpos}}\left(\pi(1\!:\!T)\right)\,;\ E_{\mathrm{tpos}}(T) \right] \in \mathbb{R}^{B\times T\times c},$$

where E_{tpos} denotes the target-position embedding and c is the conditioning dimension. The role of C is to provide explicit positional conditioning for the subsequent AdaLN modulation, while $h^{(0)}$ carries the token and sequence-order information. Thus, the backbone receives both the reordered discrete spectrum and the corresponding target-position signal in aligned tensor form.

D.3. Conditioned Transformer Propagation

Each Diffusion Transformer block contains a RMSNorm, a multi-head self-attention (with Q/K normalization) , and an MLP branch. Both branches are modulated and gated by AdaLN with condition C . We define

$$\mathrm{mod}(x;\ \Delta s, \Delta y) = x \odot (1 + \Delta y) + \Delta s.$$

For the ℓ -th layer,

$$(\Delta s_{\mathrm{msa}},\ \Delta y_{\mathrm{msa}},\ g_{\mathrm{msa}},\ \Delta s_{\mathrm{mlp}},\ \Delta y_{\mathrm{mlp}},\ g_{\mathrm{mlp}}) = \mathrm{AdaLN}(C),$$

and the hidden state update is

$$h \leftarrow h + g_{\mathrm{msa}} \odot \mathrm{MSA}\big(\mathrm{mod}(\mathrm{RMSNorm}(h);\ \Delta s_{\mathrm{msa}}, \Delta y_{\mathrm{msa}})\big),$$

$$h \leftarrow h + \mathrm{gmlp} \odot \mathrm{MLP}\big(\mathrm{mod}(\mathrm{RMSNorm}(h);\ \Delta s_{\mathrm{mlp}}, \Delta y_{\mathrm{mlp}})\big).$$

D.4. Final Layer and Decoders

Final layer first applies RMSNorm and then a last AdaLN modulation:

$$h^\star = \mathrm{mod}\big(\mathrm{RMSNorm}(h);\ \Delta s_f, \Delta y_f\big) = \mathrm{RMSNorm}(h) \odot (1 + \Delta y_f) + \Delta s_f.$$

The token (pretraining) head is

$$Z = \mathrm{LMHead}(h^\star) \in \mathbb{R}^{B\times(T)\times V}.$$

At inference, per-digit argmax gives $\hat{d}_j^{(10^3)}, \hat{d}_j^{(10^2)}, \hat{d}_j^{(10^1)}, \hat{d}_j^{(10^0)}$, and the quantized flux is reconstructed by

$$\hat{f}_j = 1000\, \hat{d}_j^{(10^3)} + 100\, \hat{d}_j^{(10^2)} + 10\, \hat{d}_j^{(10^1)} + \hat{d}_j^{(10^0)}.$$

The physic (finetuning) head is a lightweight MLP

$$\mathrm{Linear}(256{\to}256)\ \to\ \mathrm{ReLU}\ \to\ \mathrm{Dropout}(0.1)\ \to\ \mathrm{Linear}(256{\to}3)$$

whose output is

$$\boldsymbol{\theta} = [\, T_{\mathrm{eff}},\ \log g,\ [\mathrm{Fe/H}]\,] \in \mathbb{R}^{B\times 3}.$$

D.5. Loss Design

For token prediction we use focal loss :

$$\mathcal{L}_{\mathrm{FL}} = \frac{1}{BT} \sum_{b=1}^{B} \sum_{t=1}^{T} \alpha_{y_{b,t}} \left(1 - p_{b,t}(y_{b,t})\right)^{\gamma} \left(-\log p_{b,t}(y_{b,t})\right),$$

where $y_{b,t}$ is the target token at position t of sample b , $p_{b,t}(k) = \mathrm{softmax}(Z_{b,t})_k$, α_y is an optional class weight, and $\gamma \geq 0$ controls how strongly the loss focuses on hard examples.

This objective is used in the pretraining stage, where digit-wise discretisation converts spectral reconstruction into a token-level multi-class prediction problem. In this setting, the token distribution is highly imbalanced. Long spectral sequences contain many easy or high-frequency tokens, including continuum-dominated positions and padding-related tokens. In addition, digits such as $\{0, 1\}$ may appear much more frequently than other digits at high-order decimal positions. If plain cross-entropy is used, these easy tokens can dominate the total loss, while rare digit patterns and spectrally informative positions receive relatively weak supervision.

The focal modulation term $\left(1 - p_{b,t}(y_{b,t})\right)^{\gamma}$ reduces the contribution of high-confidence easy predictions and increases the relative importance of difficult tokens. This property is useful for low-SNR spectra. Around absorption-line cores, line wings, and noise-dominated regions, the digit-wise targets can be more difficult to predict because small flux perturbations may change one or more decimal digits. These positions are often more informative for learning spectral structure, but they are also more vulnerable to noise. By emphasizing hard tokens, focal loss provides stronger token-level supervision for such rare and noise-sensitive spectral regions.

This loss is used only for the symbolic reconstruction objective. The downstream stellar-parameter estimation task is treated separately as a continuous regression problem and is optimized with mean squared error in the standardized label space. Therefore, focal loss should not be interpreted as a replacement for regression loss, but as a pretraining-stage mechanism for improving supervision on imbalanced, rare, and noise-sensitive spectral tokens. Exploring hybrid token-level objectives that combine focal modulation with calibration-oriented losses is left for future work.

D.6. Masked-diffusion View and Reconstruction Metrics

In pretraining, we also view the model as an Any-Order GPT with masked-diffusion style objectives. Here we only record the exact formulas; the conceptual discussion is given in the model-architecture section of the main text.

Before entering the model, each normalized position flux f_n is quantized and decomposed into four digits:

$$q_n \ = \ \min\!\Big(9999,\ \max\!\big(0,\ \lfloor 9999\, f_n \rfloor\big)\Big), \qquad d_n^{(10^k)} \ = \ \left\lfloor \frac{q_n}{10^k} \right\rfloor \bmod 10, \quad k \in \{0,1,2,3\}.$$

The symbolic sequence can be written as

$$y = \big[\mathtt{BOS},\ d_1^{(10^3)},\ d_1^{(10^2)},\ d_1^{(10^1)},\ d_1^{(10^0)},\ \ldots,\ \mathtt{SEP},\ \ldots \mathtt{EOS}\big].$$

During training we also monitor reconstruction in the position domain. With digit-level predictive distribution p_θ ,

$$\hat{q}_n \;=\; \sum_{k=0}^{3} 10^k \sum_{r=0}^{9} r\, p_\theta\Big(d_n^{(10^k)} = r \,\Big|\, y_{\pi(<t)},\, C\Big), \qquad \hat{f}_n \;=\; \frac{\hat{q}_n}{9999}, \quad \mathrm{RMSE}_{\mathrm{flux}} \;=\; \sqrt{\frac{1}{N}\sum_{n=1}^{N}\big(\hat{f}_n - f_n\big)^2}.$$

The any-order classification objective can be written as

$$\begin{aligned} \mathcal{L}_{\mathrm{AO}} &= \mathbb{E}_{\pi\sim\mathcal{P}}\left[\sum_{t\in\mathcal{I}} w_t \cdot \mathrm{CE}\Big(y_{\pi(t)},\, p_\theta\big(\cdot \mid y_{\pi(<t)}, C\big)\Big)\right], \\ &\text{where } \pi \sim \begin{cases} \mathrm{Uniform}(\mathfrak{S}_G), & \text{with probability } p, \\ \text{AR order}, & \text{with probability } 1-p, \end{cases} \qquad \pi = \mathrm{lift}(\sigma). \end{aligned} \tag{15}$$

In the GPT in any order notation, the embedding and conditioning tensors can be equivalently written as

$$\begin{aligned} h^{(0)} &= \big[\; E_{\mathrm{tok}}(y_{\pi(1:T)})\big] \;+\; \big[E_{\mathrm{pos}}(0);\; E_{\mathrm{pos}}(\pi(1\,:\,T))\big] \in \mathbb{R}^{B\times(T)\times d}, \\ C &= \big[E_{\mathrm{tpos}}(\pi(1\,:\,T));\; E_{\mathrm{tpos}}(T)\big] \in \mathbb{R}^{B\times(T)\times c}, \end{aligned}$$

and one Diffusion-Transformer layer update can be written as

$$\begin{aligned} x_{l+\frac{1}{2}} &= x_l + \mathrm{gate}_l^{\mathrm{attn}} \cdot \mathrm{Attn}\big(\mathrm{mod}(\mathrm{RMSNorm}(x_l);\, \Delta_l^{\mathrm{attn}}, s_l^{\mathrm{attn}})\big), \\ x_{l+1} &= x_{l+\frac{1}{2}} + \mathrm{gate}_l^{\mathrm{mlp}} \cdot \mathrm{MLP}\big(\mathrm{mod}(\mathrm{RMSNorm}(x_{l+\frac{1}{2}});\, \Delta_l^{\mathrm{mlp}}, s_l^{\mathrm{mlp}})\big), \end{aligned}$$

where $(\Delta, s, \mathrm{gate})$ are linear projections of C .

For completeness, the focal-loss variant used in our backbone view over valid positions $\mathcal{I}$ is

$$\mathcal{L}_{\mathrm{focal}} = \frac{1}{|\mathcal{I}|}\sum_{i\in\mathcal{I}} \alpha(1-p_i)^{\gamma}\, \mathrm{CE}_i,$$

and the iterative "predict-unmask" denoising schedule can be written as

$$\mathcal{M}_{\tau+1} \;=\; \mathrm{TopK}_{k(\tau)}\Big(1 - p_\theta\big(y_t \mid \hat{y}^{(\tau)}, C\big)\Big), \qquad \hat{y}_t^{(\tau+1)} \;=\; \begin{cases} \arg\max_r \; p_\theta\big(r \mid \hat{y}^{(\tau)}, C\big), & t \in \mathcal{M}_\tau, \\ \hat{y}_t^{(\tau)}, & \text{otherwise.} \end{cases}$$

D.7. Optimization and Learning-rate Schedule

We use AdamW (betas $= (0.9, 0.95)$, weight_decay $= 0.01$, eps $= 10^{-8}$) with a warmup-cosine learning-rate schedule. Let the total step count be $S = E \times N_{\mathrm{iter}}$ and warmup length $S_w = 0.15\,S$; denote the base learning rate by η_0 and the minimum by $\eta_{\min} = 0.01\,\eta_0$. Then for step s ,

$$\eta(s) = \begin{cases} \eta_0\, \dfrac{s}{S_w}, & s \le S_w, \\[2ex] \eta_{\min} + \dfrac{\eta_0 - \eta_{\min}}{2}\left[1 + \cos\left(\pi\, \dfrac{s - S_w}{S - S_w}\right)\right], & s > S_w. \end{cases}$$

During pretraining, each forward pass uses a causal mask and either a random group permutation (probability $r = 0.8$) or AR order (probability $1 - r$) , designed to minimize the flux RMSE, thereby enabling the model to learn the spectral structure.

E. Finetune

We keep the input space, vocabulary, and sequence organization identical to pretraining, and only attach a lightweight regression head on top of the shared decoder-style backbone to map the sequence representation to physical parameters $\theta = [T_{\mathrm{eff}}, \log g, [\mathrm{Fe/H}]]$. This "frozen interface, minimal head change" approach aligns with empirical guidelines for scaling pretrain-finetune pipelines, enabling stable task adaptation without disrupting learned general representations[8].

E.1. Data Flow and Hierarchical Processing

Let $x_{1:T}$ denote the discrete token sequence as in pretraining, and $h_t^{(L)} \in \mathbb{R}^d$ the hidden state at layer L . We remove structure-only markers and define

$$\mathcal{V} = \{\, t \mid x_t \notin \{\texttt{<BOS>}, \texttt{<SEP>}, \texttt{<EOS>}\} \,\}, \qquad m_t = \mathbf{1}[t \in \mathcal{V}],$$

then obtain the sequence-level representation via masked mean pooling:

$$f = \frac{\sum_{t=1}^{T} m_t\, h_t^{(L)}}{\sum_{t=1}^{T} m_t} \in \mathbb{R}^d.$$

Mean pooling is a robust and comparable choice for both sentence and sequence embeddings[7].The pooled vector f is passed to a two-layer MLP head with nonlinearity and dropout:

$$\hat{y} = W_2\,\mathrm{Dropout}\big(\mathrm{ReLU}(W_1 f + b_1)\big) + b_2 \in \mathbb{R}^3.$$

To mitigate scale mismatch among targets, we compute dataset statistics $\mu, \sigma \in \mathbb{R}^3$ and standardize the targets:

$$y = \frac{\theta - \mu}{\sigma}, \qquad \hat{\theta} = \hat{y} \odot \sigma + \mu,$$

where $\odot$ denotes element-wise multiplication. Standardizing targets stabilizes quadratic losses and improves efficiency in multi-task, multi-scale regression[5].

E.2. Objective and Optimization Schedule

We minimize mean squared error in the standardized space for a batch size B :

$$\mathcal{L}_{\mathrm{MSE}} = \frac{1}{B} \sum_{i=1}^{B} \left\| \hat{y}^{(i)} - y^{(i)} \right\|_2^2.$$

We use AdamW for optimization to decouple weight decay and improve generalization[6]. The learning-rate schedule combines linear warmup with cosine annealing. With total steps S , warmup ratio ρ , warmup steps $S_w = \rho S$, base LR η_0 , and minimum LR $\eta_{\min} = \kappa \eta_0$, the step-s LR follow the exact same configuration as described before."

E.3. Evaluation and De-standardization

At inference, we de-standardize the head outputs via

$$\hat{\theta} = \hat{y} \odot \sigma + \mu,$$

and report per-parameter root mean squared error

$$\mathrm{RMSE}_k = \sqrt{\frac{1}{N} \sum_{i=1}^{N} \left(\hat{\theta}_k^{(i)} - \theta_k^{(i)} \right)^2}, \qquad k \in \{\mathrm{T_{eff}}, \log g, [\mathrm{Fe/H}]\}.$$

Keeping the same input representation and sequence organization as in pretraining helps reduce instability from representation drift. On top of that, masked mean pooling plus a minimal regression head transfers learned segment structure and noise statistics to end-to-end estimation of physical parameters[7].

E.4. From Pretrained Representations to Physical-parameter Regression

We keep exactly the same input space, vocabulary, and sequence layout as in pretraining (i.e., the same tokenization; sequences begin with `[BOS]`, end with `[EOS]`, and are right-padded with `[SEP]`) . On top of the shared encoder we only attach a regression head. This design aims to transfer the pretrained representation of a "spectrum that can be completed" to physical estimation with *minimal change*, which is suitable for small labeled datasets and cross-instrument scenarios.

We adopt this finetuning setup because the pretraining task of *spectral completion* has already driven the backbone encoder to capture the continuum shape and the covarying structure of line families that are tied to T_{eff} , $\log g$, and $[\mathrm{Fe/H}]$. On this basis, continuing to use the *same* input space and sequence organization avoids the *domain-shift* instability that would arise from changing the representation.

Early stopping and model selection use the *minimum validation loss*. For evaluation and reporting we work on the physical scale. We then report the per-parameter errors and an aggregate score which facilitates like-for-like comparison across experimental setups.

F. Supplement to the Results Section

Here we provide the details of the figures cited in the results section of the main text (See Fig. 1,Fig. 2,Fig. 3,Fig. 4 and Fig.5).

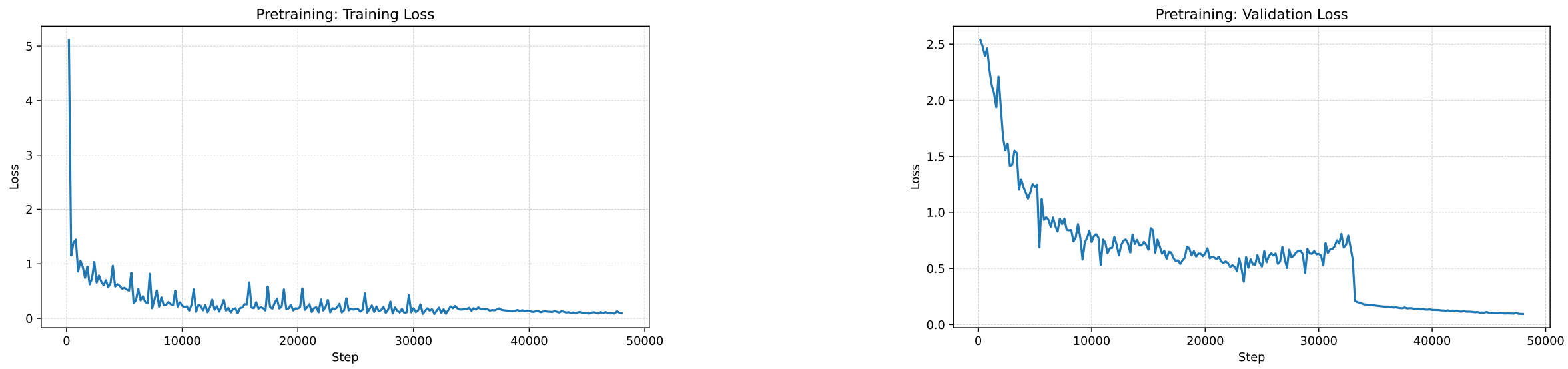

Figure 1: Pretraining dynamics (Loss). Left: training loss versus optimization steps; right: validation loss. The checkpoint with the minimum validation loss is selected for subsequent finetuning.

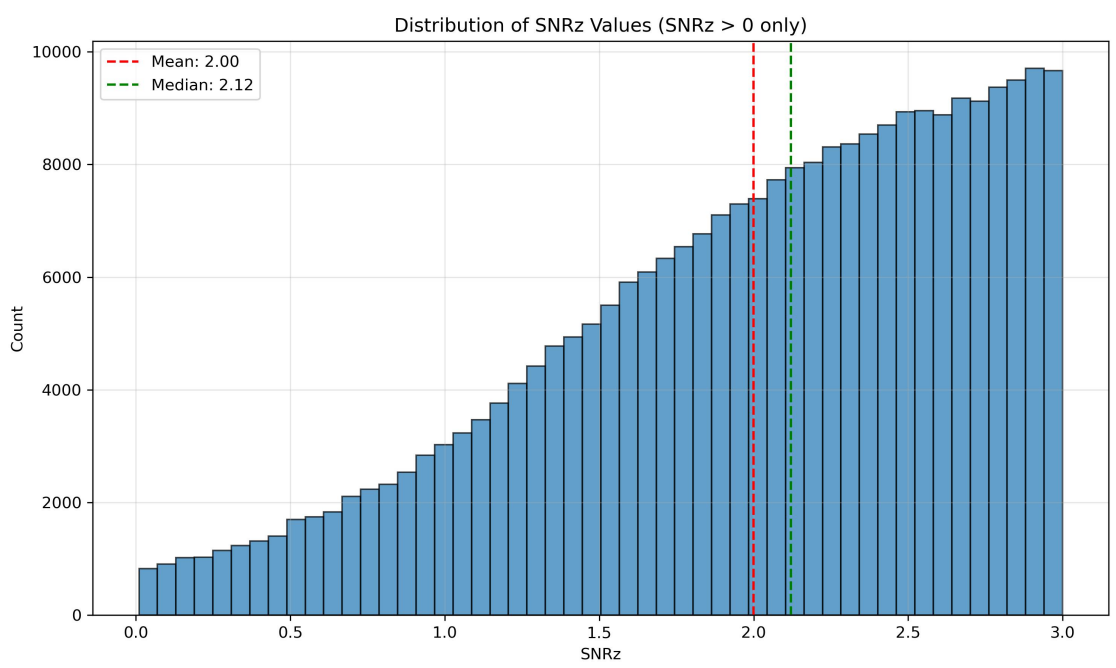

Figure 2: The SNR-z distribution for LAMOST LRS stellar spectra.

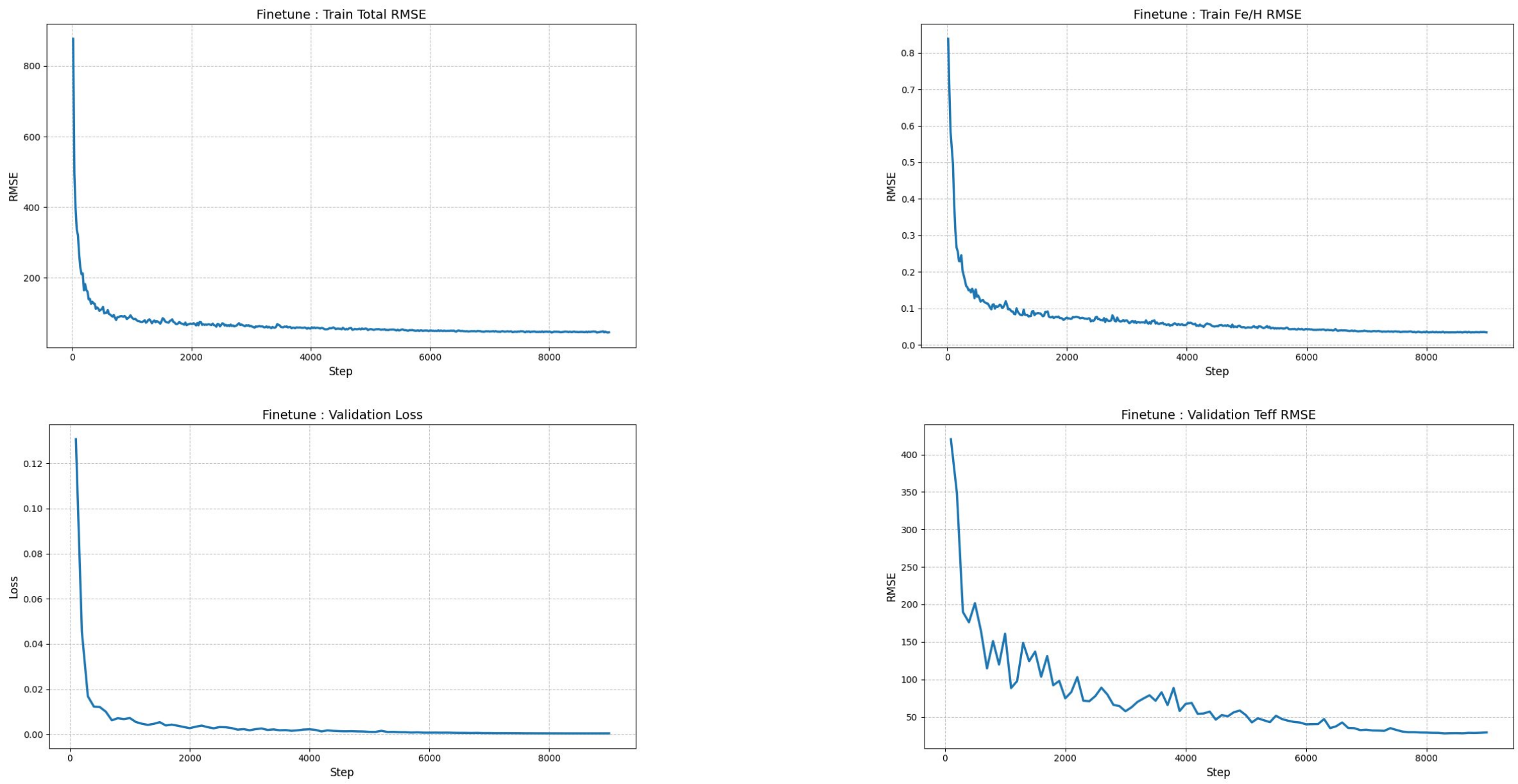

Figure 3: Finetuning diagnostics on high-SNR synthetic spectra (Part I). Each panel plots a scalar metric against training step.

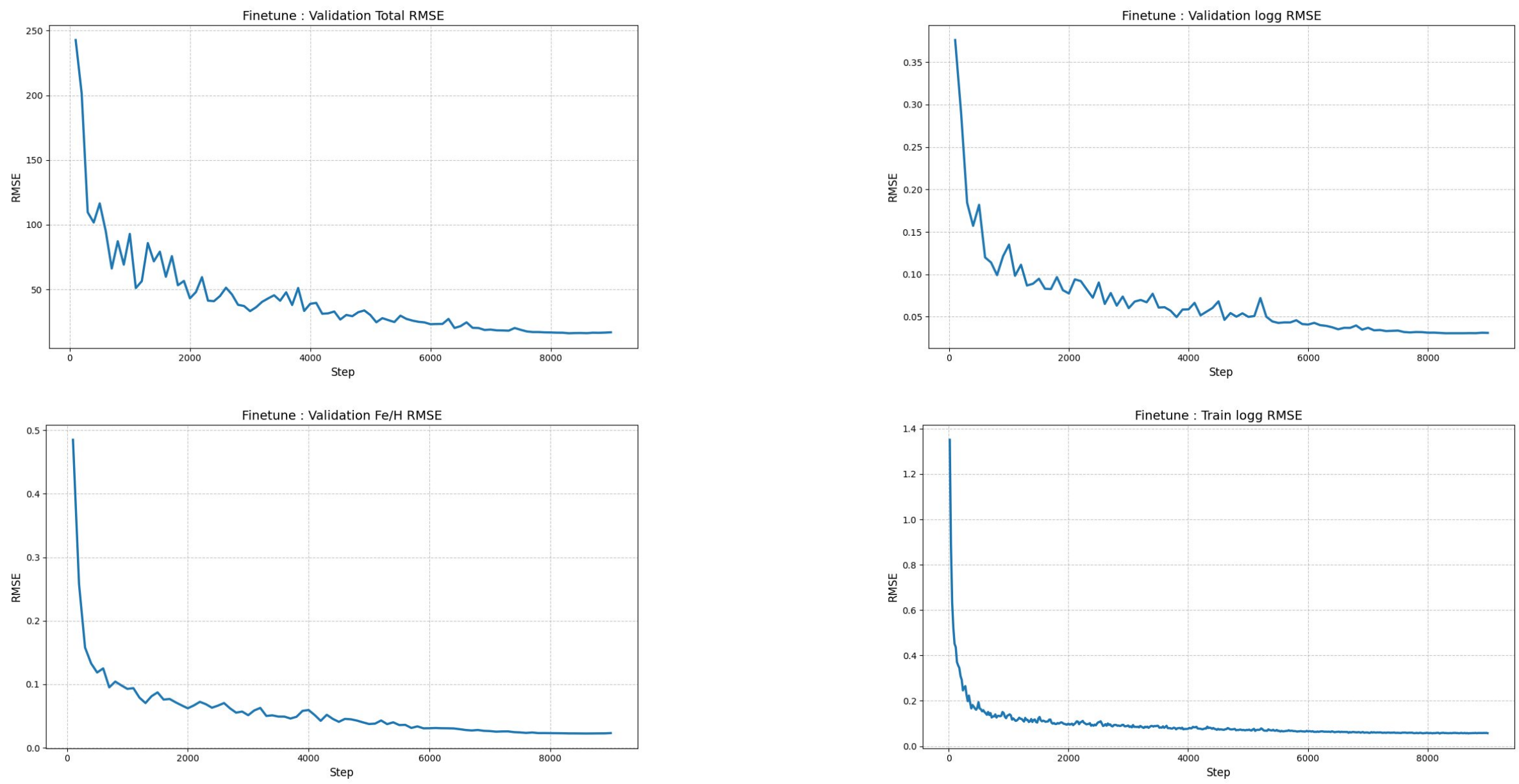

Figure 4: Finetuning diagnostics on high-SNR synthetic spectra (Part IIa). Validation total RMSE, validation $\log g$ RMSE, validation [Fe/H] RMSE, and training $\log g$ RMSE during downstream optimization.

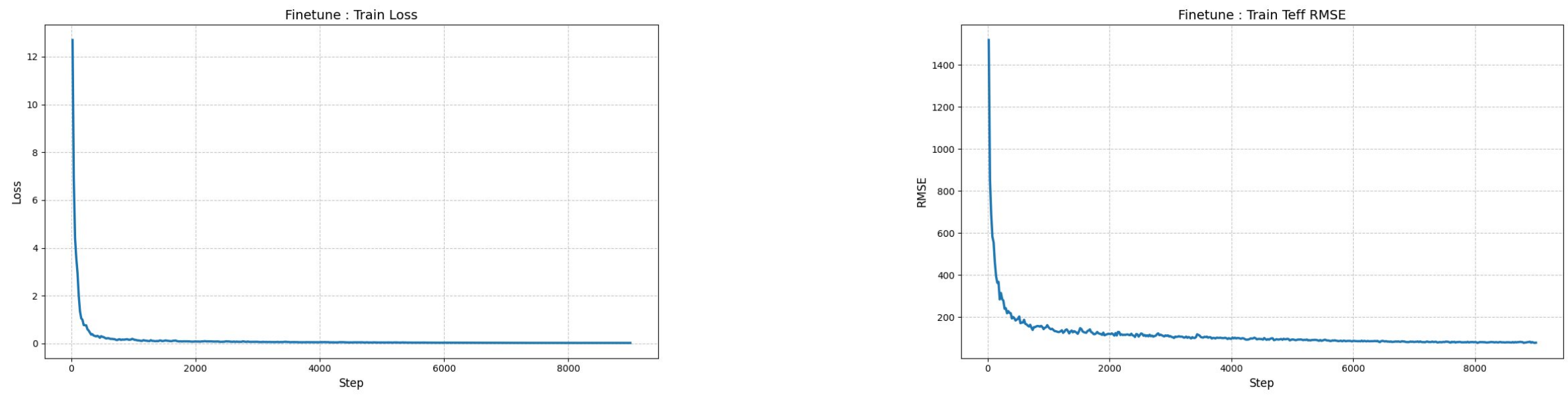

Figure 5: Finetuning diagnostics on high-SNR synthetic spectra (Part III). Training loss and training $T_{\rm eff}$ RMSE during downstream optimization.

References

[1] Aoki, W., Li, H., Matsuno, T., et al., 2022. Four-hundred very metal-poor stars studied with lamost and subaru. i. survey design, follow-up program, and binary frequency. The Astrophysical Journal 931, 146. URL: https://dx.doi.org/10.3847/1538-4357/ac6515, doi:10.3847/1538-4357/ac6515.

[2] Bratsolis, E., Sigelle, M., 2001. A spatial regularization method preserving local photometry for richardson-lucy restoration*. A&A 375, 1120–1128. URL: https://doi.org/10.1051/0004-6361:20010709, doi:10.1051/0004-6361:20010709.

[3] Hou, X., Zhao, G., Li, H., 2024. Very metal-poor stars i: a catalogue derived from lamost dr9. Monthly Notices of the Royal Astronomical Society 532, 1099–1111. URL: https://doi.org/10.1093/mnras/stae1567, doi:10.1093/mnras/stae1567, arXiv:https://academic.oup.com/mnras/article-pdf/532/1/1099/58425974/stae1567.pdf.

[4] Howell, S.B., 2006. Handbook of CCD Astronomy. Cambridge Observing Handbooks for Research Astronomers. 2 ed., Cambridge University Press.

[5] Kendall, A., Gal, Y., Cipolla, R., 2018. Multi-task learning using uncertainty to weigh losses for scene geometry and semantics, in: Proceedings of the IEEE conference on computer vision and pattern recognition, pp. 7482–7491.

[6] Loshchilov, I., Hutter, F., 2017. Decoupled weight decay regularization. arXiv preprint arXiv:1711.05101 .

[7] Reimers, N., Gurevych, I., 2019. Sentence-bert: Sentence embeddings using siamese bert-networks. arXiv preprint arXiv:1908.10084 .

[8] Tay, Y., Dehghani, M., et al., 2021. Scale efficiently: Insights from pre-training and fine-tuning transformers. arXiv preprint arXiv:2109.10686 .
